# Combining Electrochemical Reduction with Biosynthesis for Directed Conversion of CO_2_ into a Library of C3 Chemicals

**DOI:** 10.1002/advs.202522097

**Published:** 2026-01-04

**Authors:** Kaixing Xiao, Shanquan Liang, Xujun Zhao, Zhiyao Peng, Ruoshi Luo, Jikai Zong, Ling Zhou, Yude Su, Dan Wang

**Affiliations:** ^1^ Department of Chemical Engineering School of Chemistry and Chemical Engineering Chongqing University Chongqing China; ^2^ Suzhou Institute for Advanced Research University of Science and Technology of China Suzhou Jiangsu China; ^3^ State Key Laboratory of Coal Mine Disaster Dynamics and Control Chongqing University Chongqing China

**Keywords:** biosynthesis, C3 chemicals, carbon dioxide, carbon neutrality, microbial electroreduction

## Abstract

Electrochemical carbon dioxide (CO_2_) reduction presents significant opportunities for sustainable chemical manufacturing. However, conventional electrochemical CO_2_ reduction typically produces only C1 or C2 products, and the direct synthesis of C3 chemicals remains a major challenge. In this study, we developed a unique tandem system integrating microbial electroreduction with biosynthesis, demonstrating the feasibility of using CO_2_‐derived acetic acid as a carbon source for biosynthesis. In Module I, taking advantage of an established perfluorocarbon nanoemulsion strategy for enhanced H_2_ delivery, we achieved efficient electrocatalytic CO_2_‐to‐acetate conversion with an acetic acid production rate of 0.34 ± 0.01 g L^−1^ day^−1^. In Module II, the engineered was established by developing an ultrahigh mutation system to facilitate the screening of desirable microbes with high toxicity tolerance to acetic acid and different products. Various C3 chemicals were efficiently synthesized by this tandem system, including β‐alanine (2.1 g/L), acrylic acid (752.4 mg/L), and L‐lactic acid (672.9 mg/L), achieving a conversion yield of 0.54–0.72 tons of destined products per ton of CO_2_ with an energy consumption of only 51.88–80.7 GJ. This study introduced a novel approach for upcycling CO_2_ into a library of high‐value C3 chemicals, offering a new approach for achieving carbon neutrality.

## Graphical abstract

In the H‐type electrolytic cell, carbon dioxide is reduced to acetic acid via electro‐microbial catalysis. The simply processed acetic acid is further converted through biological fermentation into high‐value‐added products, including acrylic acid, L‐lactic acid, and β‐alanine.

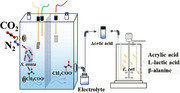

**Abbreviations**
ACAL: β‐alanyl‐CoA: ammonia lyase 2
ACH: Acyl‐CoA thioester hydrolase
ACS: Acetyl‐CoA by acetyl‐CoA synthetase
ACT: β‐alanine CoA‐transferase
ADC: Aspartate 1‐decarboxylase
AEM: Anion exchange membrane
AH: Aconitate hydratase B
ALE: Adaptive laboratory evolution
AspA: Aspartate ammonia‐lyase
CS: Citrate synthase
DLDH: D‐lactate dehydrogenase
ECRR: Electrocatalytic CO_2_ reduction reaction
FH: Fumarate hydratase
ICL: Isocitrate lyase
LLDH: L‐lactate dehydrogenase
MDH: Malate dehydrogenase
MES: Microbial electrosynthesis
PHB: Polyhydroxybutyrate
SDH: Succinate dehydrogenase
TCA: Tricarboxylic acid
UMS: Ultrahigh mutation system

## 1 Introduction

The excessive emission of CO_2_ is one of the main contributors to the greenhouse effect [1, 2]. Conversion of CO_2_ into value‐added chemicals has emerged as a promising strategy to address this issue [3, 4, 5]. Electrochemical reduction represents a pivotal approach for artificially transforming CO_2_ into high value‐added chemicals due to its ambient reaction conditions, flexible system design, environmental friendliness, and the use of renewable electricity [6, 7, 8, 9]. However, existing electrochemical CO_2_ reduction technologies suffer from two limitations that impede its development toward industrial‐level applications. On the one hand, due to the multiple proton‐coupled electron transfer process, CO_2_ reduction typically produces diverse carbon products (e.g., carbon monoxide (CO), methanol (CH_3_OH), and formic acid (HCOOH)) with H_2_ evolution as the competing reaction. On the other hand, the products obtained from traditional electrochemical CO_2_ reduction are typically limited to C1 and C2 compounds, and directly production of high‐value C3 and C3+ chemicals from CO_2_ remains a major challenge [10].

Despite the limited product spectrum, the C1 and C2 compounds (e.g., CH_3_OH, HCOOH) produced in electrochemical CO_2_ reduction can serve as substrates for biosynthetic fermentation. This association has motivated the development of cascade systems by combining electrochemical CO_2_ reduction with biosynthesis, which shows an attractive pathway for converting CO_2_ into longer‐chain compounds. For example, Zhou et al. used Pd‐Cu aerogel to electrocatalytically reduce CO_2_ to CH_3_OH, followed by a biocatalytic system to oxidize methanol to ethylene glycol, glycolic acid, and D‐erythrose through the employment of glycolaldehyde synthase and alcohol oxidase [11]. Zheng et al. described a hybrid electro‐biosystem coupling spatially separate CO_2_ electrolysis with yeast fermentation, which enabled efficient CO_2_ conversion into glucose [12]. Lee et al. proposed a two‐step abiotic‐biotic system in which CO_2_ was converted into acetate via a Cu‐Ag series electrode. The electrosynthesized acetate was then fed into a bioreactor and biopolymerized by *Cupriavidus necator* to produce 3‐hydroxybutyrate [13]. Chen et al. developed an electro‐biodiesel concept that leverages electrocatalytic CO_2_ reduction reaction (ECRR) to produce C2+ intermediates with subsequent microbial conversion of these intermediates into lipids as biodiesel [14]. Despite this research progress, existing electro‐microbial systems typically employ inorganic electrocatalysts to perform the upstream ECRR, which inevitably produce byproducts with potential bio‐toxicity in addition to the target intermediate. This results in a time‐consuming and costly purification process of the target intermediate before being used as feedstock for the downstream engineered biocatalysis. Recently, microbial CO_2_ electroreduction technology has emerged as a promising alternative to conventional inorganic‐based ECRR systems. In such systems, electrons are transferred from the cathode directly or via a mediator to CO_2_‐reducing microorganisms that are situated on or near the electrode surface, and these microorganisms reduce CO_2_ into specific C1 or C2 products via their internal metabolic processes. Owing to the specific metabolic pathway of CO_2_‐reducing microorganisms, this process enables the production of high‐purity C1 or C2 products (e.g., acetic acid, methane) at the cathode, which can be readily separated and used as carbon sources for downstream biosynthesis. For example, Su et al. developed a microbial CO_2_ electroreduction system by integrating bacteria *Sporomusa ovata* with high‐surface‐area silicon nanowire electrodes, which converts CO_2_ into high‐purity acetate with an impressive solar‐to‐acetate production with 3.6% efficiency [15]. Liu et al. reported that the acetic acid produced from microbial CO_2_ electroreduction can be directly utilized for the biosynthesis of amorphadiene, butanol, PHB, isobutanol, butanol and hexanol by genetically engineered *E. coli* [16]. Despite these advances, the synergetic effect of combined microbial electroreduction with biosynthesis remains largely underexplored. The synthesis of a range of value‐added platform chemicals, such as acrylic acid and L‐lactic acid via a combined strategy has not yet been demonstrated.

Acetic acid has garnered significant interest as a promising fermentation feedstock due to its high solubility, bioavailability, and carbon‐negative production routes [17]. By incorporating acetic acid into the bacteria metabolic pathway through acetyl‐CoA intermediate, a large variety of products can be produced with high selectivity [18]. A wide range of longer‐chain acid products, such as malate, succinate have been demonstrated using acetic acid as a carbon feed source [19]. Additionally, higher‐value products such as acetoin and 2,3‐Butanediol have also been produced from engineered *E. coli* strains at > 25% of the theoretical titers through selective breeding and genetic modification [18]. Among all possible products from bacterial fermentation using acetic acid as the substrate, acrylic acid, lactic acid, and β‐alanine attract particular interest due to their distinct properties and widespread applications. Acrylic acid, mainly used in superabsorbent polymers, is traditionally synthesized from oil‐derived propylene via two‐step oxidation, an energy‐intensive process with potential explosion risk [20]. Lactic acid, a cornerstone of biodegradable polylactic acid plastics, is mainly produced through microbial fermentation of starch or sucrose‐rich biomass [21]. Nevertheless, this approach relies on food‐competing feedstocks and involves complex downstream purification that requires high energy and chemical consumption. β‐alanine, an amino acid used in food and pharmaceuticals, is conventionally manufactured through chemical synthesis from ammonia and petroleum‐derived precursors under harsh conditions, or through fermentation using glucose as substrates [22]. However, these traditional production pathways all face challenges of high carbon footprints, operational complexity, and dependence on either fossil fuels or edible biomass. From a global perspective, the annual market demand reaches approximately 9 million tons for acrylic acid [20], 1.96 million tons for lactic acid [23], and 81,000 tons for β‐alanine [24]. This highlights the urgency for seeking alternative sustainable synthesis strategies from non‐food raw materials such as CO_2_.

**FIGURE 1:**
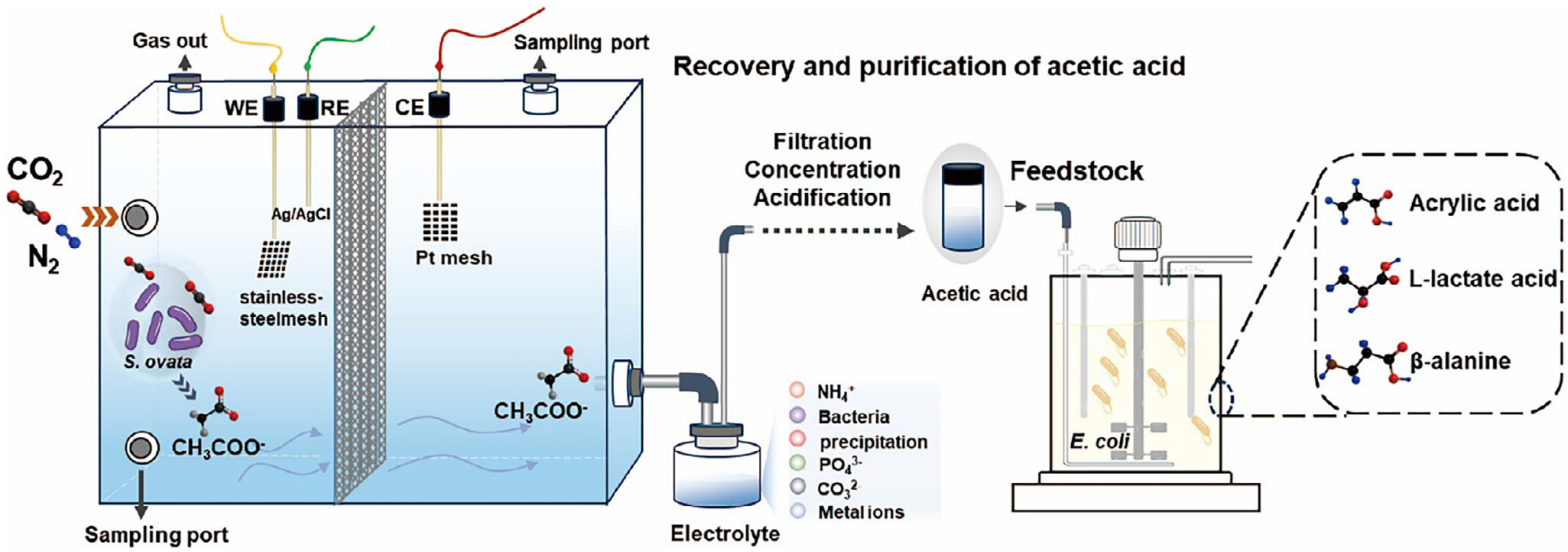
Microbial electroreduction and biosynthesis combining system for sustainable upgrading CO_2_ into high value C3 chemicals.

In this study, we developed a microbial electroreduction‐biosynthesis tandem platform that enabled the directed conversion of CO_2_ into a library of high‐value C3 chemicals (Figure 1). This system consisted of a microbial CO_2_ electroreduction module (module I) and a microbial biosynthesis module (module II). By employing a perfluorocarbon nanoemulsion strategy for enhanced H_2_ delivery, the microbial CO_2_ electroreduction module achieves efficient acetate production in a 1 L upscaled electrolyzer, with a production rate of 0.34 ± 0.01 g L^−^^1^ day^−^^1^ and a Faradaic efficiency of 71.46 ± 2.75%. Compared to the reported electro‐microbial hybrid catalytic systems, CO_2_‐derived acetic acid was simply separated and concentrated from the electrolyte in our approach, which subsequently allowed microbial fermentation to be conducted with the precise concentration of the CO_2_‐derived acetic acid being added on demand [16]. Through meticulous synthetic biology design, this system achieved targeted synthesis of three kinds of C3 platform chemicals from CO_2_, including β‐alanine (titer up to 2.1 g/L), acrylic acid (titer up to 752.4 mg/L), and L‐lactic acid (titer up to 672.9 mg/L). Additionally, a novel ultra high mutation system was developed to facilitate the screening of microbes, and engineered strains with tolerance to high concentrations of acetic acid and acrylic acid were obtained. This work validated the feasibility of using CO_2_ derived acetic acid as a carbon source for biosynthesis, diversifying the product portfolio of the microbial electroreduction‐biosynthesis system, and demonstrating a novel approach for producing bulk chemicals, distinct from conventional petroleum‐based routes.

Within the *S. ovata* strain, acetate was synthesized via the Wood‐Ljungdahl pathway. The acetate then migrates across an anion exchange membrane (AEM) into the anodic chamber. The acetate‐containing electrolyte was collected from the cathode chamber, and then the acetate‐containing electrolyte was filtered, concentrated, and acidified to obtain pure acetic acid. Finally, the purified acetic acid was introduced as a feedstock into a 5 L bioreactor for the synthesis of C3 products. WE, Working Electrode; RE, Reference Electrode; CE, Counter Electrode. CO_2_ was introduced into the microbial electrocatalytic reactor.

## 2 Results and Discussion

### 2.1 Electrochemically Catalyzed Conversion of CO_2_ to Acetate

**FIGURE 2:**
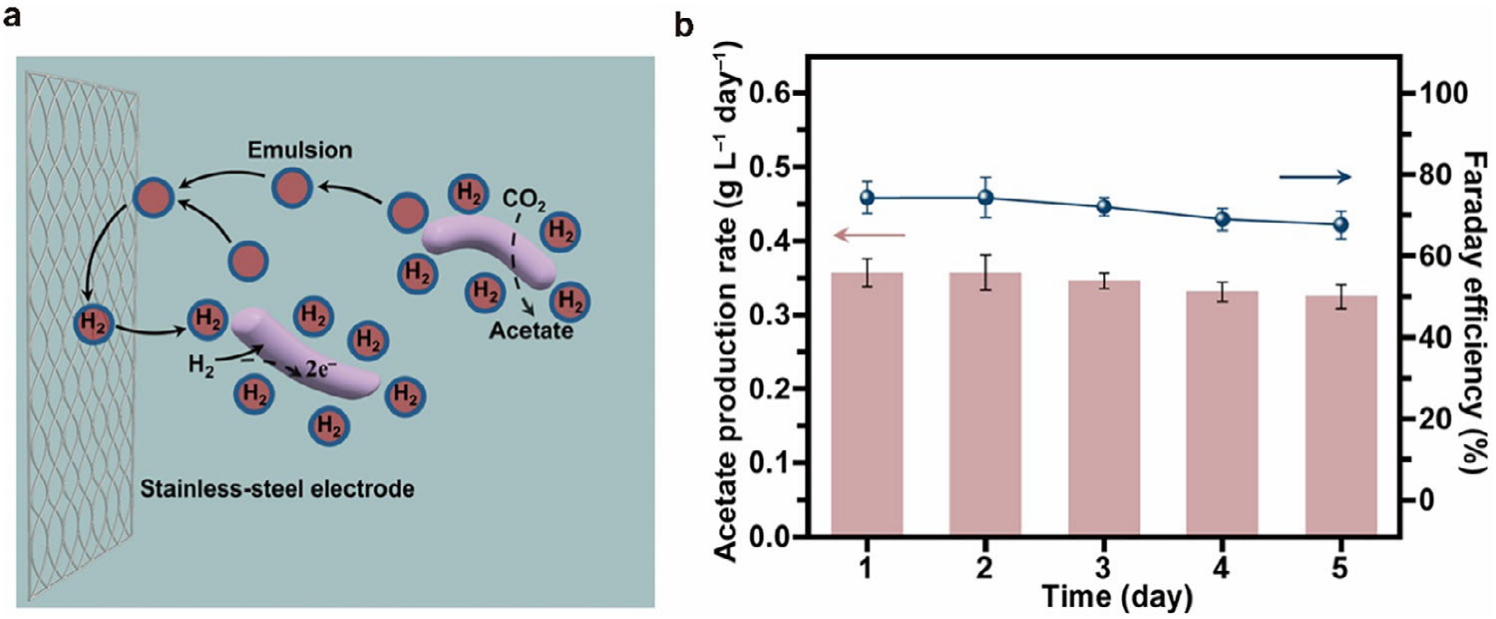
High‐purity acetic acid from CO_2_ catalyzed by electrochemical. (a) Mechanism of perfluorocarbon nanoemulsions in acetate production via MES; (b) Daily acetate production rate and Faraday efficiency in the scaled‐up MES system under galvanostatic conditions (2 mA cm^−2^) over 5 days. Reactions were performed in triplicate (n = 3 biologically independent samples) and data are presented as mean values ± SD. Source data are provided as a Source Data file.

In order to achieve efficient synthesis of high‐purity acetic acid via microbial CO_2_ electroreduction, we adopted a perfluorocarbon nanoemulsion strategy established in a previous study [14]. Specifically, we designed a scaled‐up H‐type electrolytic cell (Figure 1) with two chambers separated by an AEM. A phosphate‐enhanced DSMZ 311 medium (Table S1) was employed as the electrolyte, which enables effective stabilization of system pH under high current density. In the cathodic chamber, a Co‐P alloy‐coated stainless‐steel mesh with an area of 25 cm^2^ was employed as the working electrode to enable efficient H_2_ evolution. Perfluorocarbon nanoemulsions (5% v/v, added to the cathodic chamber) were introduced to enhance the H_2_ solubility as well as its transfer kinetics via non‐specific binding with *S. ovata*, resulting in elevated H_2_ uptake, intracellular assimilation, and thereby facilitated CO_2_ reducing rate by *S. ovata*. Consequently, the system achieved efficient acetate production with an average production rate of 0.34 ± 0.01 g L^−1^ day^−1^ and a Faradaic efficiency of 71.46 ± 2.75% at 2 mA/cm^2^ current density (Figure 2). After operating for 5 days, a total acetate titer of 1.70 g/L (corresponding to a total mass of 1.2 g) can be obtained (n = 3). While the Faradaic efficiency (71.46%) in this study was comparable to some reported electrocatalytic CO_2_‐to‐acetate systems, the distinct advantage of our microbial electrosynthesis platform lied in its integrated process benefits, as shown in Table S10. A simple, low‐cost Co‐P electrodeposited catalyst and reactor reduced the system complexity. Full biocompatibility allowed operation in microbial media for the conversion of CO_2_ to higher‐value biochemicals, which is a feature unattainable in purely electrocatalytic systems. This electrocatalytic system was characterized by high product selectivity with gaseous H_2_ as the sole by‐product and superior operational stability (> 120 h under mild conditions). This combination strategy ensures high carbon utilization and lowers separation costs by avoiding liquid organic co‐products. Thus, the innovation of this work was reflected not solely in efficiency, but in the synergistic combination of selectivity, stability, biocompatibility, and process simplicity.

Furthermore, the acetate‐containing electrolyte was centrifuged and filtered to remove biological cells and insoluble substances. Subsequently, the electrolyte was concentrated to 20% of its initial volume by solvent evaporation at 120°C, enabling precise control of acetic acid concentration based on the specific requirement of the downstream fermentation process. The pH of the concentrated solution was then adjusted to 2.0 using concentrated sulfuric acid to convert acetate to acetic acid. During this process, the cathode of the electrolytic cell contained approximately 700 mL of acetic acid‐containing electrolyte with a concentration of about 1.70 g/L. The concentration step reduced the volume to 20% of the original (approximately 140 mL), which involved evaporating 560 mL of water and required an energy consumption of 1.29 MJ. At an electricity cost of 0.05$/kW h, this translated to a batch cost of $0.018. The acidification step required 0.3 mol of sulfuric acid, adding an extra cost of $0.01 per batch. Thus, at the small scale of this study, the energy and cost associated with concentration and acidification remained low cost. The resulting acetic acid with precisely controlled concentration is ready to feed into the biosynthetic process in module II.

As a core intermediate in CO_2_ bioconversion, efficient anaerobic fixation of CO_2_ to acetic acid was achieved in this study via the Wood‐Ljungdahl pathway in *S. ovata*, providing C2 skeletons for carbon chain elongation [25]. So the next key manipulation is constructing engineered strains through metabolic engineering and synthetic biology to efficiently use this novel substrate CO_2_ derived acetic acid to produce high‐value‐added chemicals.

### 2.2 Design of the Multi‐Enzyme Cascade Pathways for C3 Compounds

**FIGURE 3:**
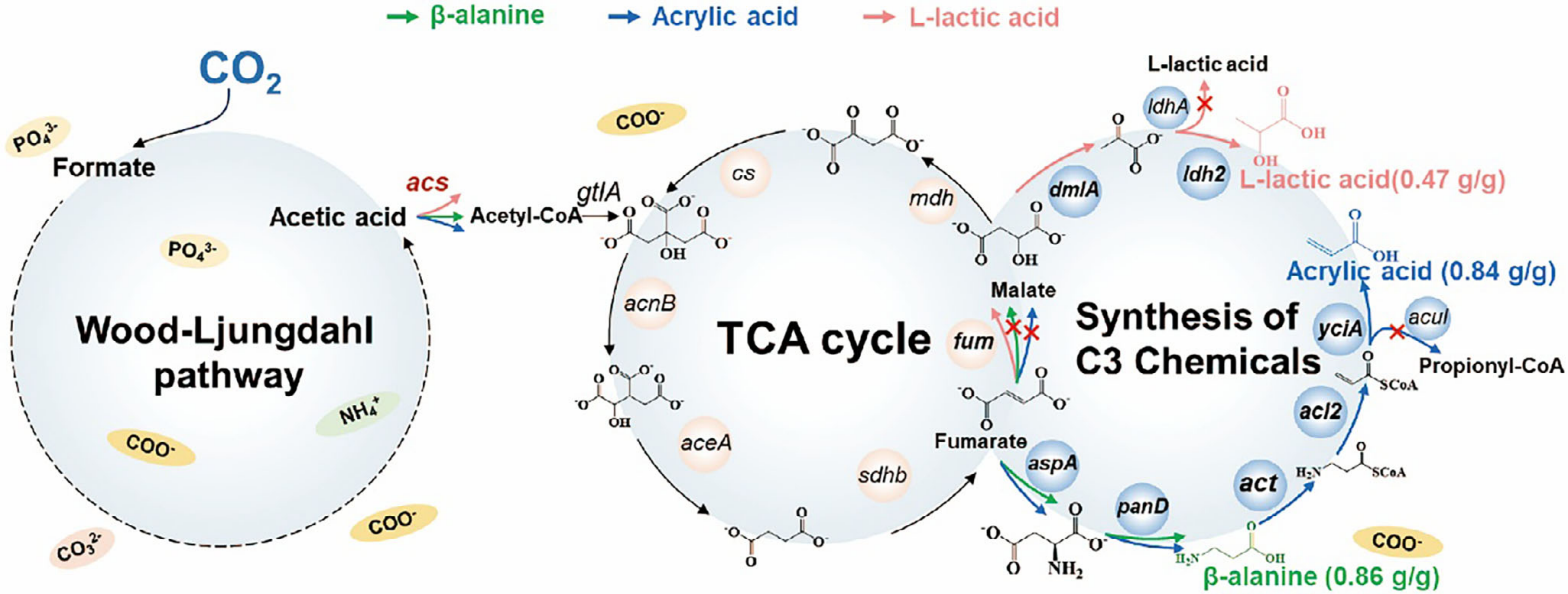
The microbial electroreduction‐biosynthesis tandem system for C3 chemicals production from CO_2_.

Nowadays, relative pure acetic acid has been produced in the MES system in our study and some other academic group, we turned our attention to a more formidable challenge: the synthesis of high‐value‐added organic acids with higher efficiency and purity. In this section, multi‐enzyme cascade pathways for efficiently utilize the novel feedstock CO_2_‐derived acetic acid for C3 organic acid production were explored. As shown in Figure 3, the constructed pathway of three organic acids, acrylic acid, L‐lactic acid, and β‐alanine, all commenced with the activation of acetic acid to acetyl‐CoA by acetyl‐CoA synthetase (ACS), acetyl‐CoA converted into citrate by citrate synthase (CS), then entering into the tricarboxylic acid (TCA) cycle. For β‐alanine production, *acs* gene encoding Acetyl‐coenzyme A synthetase (ACS), *gltA* gene encoding citrate synthase (CS), *acnB* gene encoding Aconitate hydratase B (AH), *aceA* gene encoding Isocitrate lyase (ICL), *sdhb* gene encoding Succinate dehydrogenase (SDH), *aspA* gene encoding aspartate ammonia‐lyase (AspA) and *panD* encoding Aspartate 1‐decarboxylase (ADC) were overexpressed. The enzymes ACS, CS, AH, ICL, SDH, AspA, and ADC carrying a C‐terminal His‐tag in *E. coli* BL21 (DE3) were assessed by SDS‐PAGE, as shown in Figure S1. The sizes of the recombinant proteins were 72, 120, 99, 48, 27, 52, and 14 kDa respectively. ADC exhibited low activity toward aspartate, a series of high‐activity variants of the ADC enzymes were generated through site‐directed mutagenesis, whereas ADC mutations (L20E/R99T, L20G/R99T, N112E/V123D, N112A/V123D) displayed enhanced activities. The ADC L20G/R99T (ADC^#^) showed the greatest activity shown in Table S9. The activities of purified ADC and ADC^#^ toward aspartate were 3.57 and 132.24 U/mg, respectively, a 37‐fold improvement was achieved. The titer of β‐alanine was 389.25 mg/L after 12 h reaction time in 100 mm Tris‐HCl buffer (pH 7.5), with enzymes of ACS, ACl, AH, ICL, SDH, AspA, and ADC^#^ were setting at 2.0, 1.0, 1.0, 1.0, 1.0, 1.0, and 15.0 µm, respectively. For acrylic acid production, *acs*, *gltA, acnB*, *aceA*, *sdhb*, *aspA*, *panD*, act gene encoding β‐alanine CoA‐transferase (ACT), *acl2* gene encoding β‐alanyl‐CoA: ammonia lyase 2 (ACAL), and *yciA* gene Acyl‐CoA thioester hydrolase (ACH) were overexpressed. The enzymes ACT, ACAL, and ACH carrying a C‐terminal His‐tag in *E. coli* BL21 (DE3) were assessed by SDS‐PAGE, as shown in Figure S1. The sizes of the recombinant proteins were 42, 14 and 17 kDa, respectively. ACH showed significantly low activity toward acrylyl‐CoA, then series of high‐activity variants of the ACH enzymes were generated through site‐directed mutagenesis, whereas ACH mutations (D39E/S89D, D39A/S89D, D39T/S89D, D39L/S89D) displayed enhanced activities. The ACH D39T/S89D (ACH^#^) showed the greatest activity shown in Table S9. The activities of purified ACH and ACH^#^ toward acrylyl‐CoA were 0.0045 and 99.75 U/mg, respectively, a 22167‐fold improvement was achieved. The titer of acrylic acid was 30.27 mg/L after 12 h reaction time in 100 mm Tris‐HCl buffer (pH 7.5), with enzyme of ACS, ACl, AH, ICL, SDH, AspA, ADC^#^, ACT, ACAL and ACH^#^ were setting at 2.0, 1.0, 1.0, 1.0, 1.0, 1.0, 25.0, 1.0 and 10.0 µm, respectively. For L‐lactic acid production, *acs*, *gltA, acnB*, *aceA*, *sdhb*, *fumABC* encoding fumarate hydratase (FH), *dmlA* encoding malate dehydrogenase (MDH), and *ldh2* encoding L‐lactate dehydrogenase (LLDH) were overexpressed. The enzymes FH, MDH and LLDH carrying a C‐terminal His‐tag in *E. coli* BL21 (DE3) were assessed by SDS‐PAGE, as shown in Figure S1. The sizes of the recombinant proteins were 170, 40 and 37 kDa respectively. MDH was a key enzyme in the conversion of malate to lactate. However, assays showed that its catalytic activity toward malate was very low. A series of modifications were performed on MDH, whereas mutations of MDH (N135G/R183D, N135K/R183D, E99K/N293D, E99D/N293D) displayed enhanced activities. The MDH E99D/N293D (MDH^#^) showed the greatest activity shown in Table S9. The activities of purified MDH and MDH^#^ toward aspartate were 4.80 and 42.64 U/mg, respectively, a 9‐fold improvement was achieved. The titer of L‐lactic acid was 101.39 mg/L after 12 h reaction time in 100 mm Tris‐HCl buffer (pH 7.5), with the enzyme of ACS, ACl, AH, ICL, SDH, FH, MDH^#^, and LLDH were setting at 2.0, 1.0, 1.0, 1.0, 1.0, 2.0, 25.0, and 2.0 µm, respectively. This approach demonstrated a tandem process for CO_2_ conversion that synergistically couples electrochemical acetic acid production with its biocatalytic upgrade to C3 chemicals. This study opened a new avenue for synthesizing complex molecules from CO_2_, moving beyond single‐step conversion limitations.

As a further evaluation of the aforementioned C3 compound synthesis pathways, thermodynamic parameters were calculated to assess their efficiency potential for in vitro synthesis. The total Gibbs free energy changes from acetic acid to acrylic acid, β‐alanine, and L‐lactic acid were determined to be −28.8, −44.2, and −40.1 kcal/mol, respectively, as shown in Table S2. The standard Gibbs free energy change (ΔG'^0^) of the enzymatic reaction at pH 7.5 and ionic strength of 0.25 m (http://equilibrator.weizmann.ac.il/). It was indicated that all three pathways from acetic acid to acrylic acid, β‐alanine, and L‐lactic acid were thermodynamically feasible and could effectively proceed under in vitro catalytic conditions.

Compared to C1 compounds such as formate and methanol, acetic acid was more readily utilized by microorganisms due to it could be directly activated to acetyl‐CoA and subsequently entered the central metabolism, whereas formate and methanol had to be further metabolized through the RuMP pathway [26] or the serine pathway [27], involving methanol dehydrogenase [28] or formaldehyde assimilation pathways [29]. Research demonstrated that, in contrast to the TCA cycle, these pathways require substantial amounts of ATP and NADH [30]. After acetic acid was activated to acetyl‐CoA and entered the TCA cycle, more ATP and NADH could be produced [31], thereby creating advantages for microbial growth and subsequent synthesis of C3 compounds. From the perspective of product synthesis pathways, a key advantage provided by the efficient utilization of acetic acid was its facilitation of directional carbon flux regulation [32]. The synthesis of all three compounds required acetyl‐CoA to be carboxylated and extended to a C3 skeleton, a process that was strictly dependent on a high‐flux input at the acetyl‐CoA node. As a direct precursor, acetic acid avoided the positional ambiguity of carbon atoms due to carbon rearrangement during C1 substrate assimilation, and was also more conducive to being precisely directed toward target molecules [33].

The green arrows indicate the synthetic pathway for β‐alanine, the blue arrows point to the synthetic pathway for acrylic acid, and the pink arrows represent the synthetic pathway for L‐lactic acid. The *fumABC* and *acuI* genes were knocked out in the acrylic acid‐producing strain, the *ldhA* gene was knocked out in the L‐lactic acid‐producing strain, and the *fumABC* gene was knocked out in the β‐alanine‐producing strain. Genes abbreviations are encoding: *acs*, Acetyl‐coenzyme A synthetase; *gltA*, citrate synthase; *sdhb*, Succinate dehydrogenase; *fum*, fumarate hydratase; *aspA*, Aspartate ammonia‐lyase; *panD*, Aspartate 1‐decarboxylase; *act*, β‐alanine CoA‐transferase; *acl2*, β‐alanyl‐CoA:ammonia lyase; *yciA*, CoA thioesterase; *dmlA*, malate dehydrogenase; *ldhA*, D‐lactate dehydrogenase; *ldh2*, L‐lactate dehydrogenase.

### 2.3 Biosynthesis of Acrylic Acid in Engineered Strains

We established a novel biosynthetic pathway in *E. coli* for producing acrylic acid from CO_2_‐derived acetic acid. Upon the in vitro experiments results above about acrylic acid, the engineered *E. coli* strains XKX114/pET‐*acs*‐*panD*‐*act‐acl2*‐*yciA* (*panD*: RBS15000 sequence, *yciA*: RBS8000 sequence) (Table S4) was constructed to examine its acrylic acid production performance. Acetic acid was added as the substrate to a final concentration of 5 mm. A decline in the pH of the fermentation broth from 7.2 to 4.2 was observed, with a noticeable growth inhibition of the XKX114/pET‐*acs*‐*panD*‐*act‐acl2*‐*yciA* (*panD*: RBS15000 sequence, *yciA*: RBS8000 sequence) strain. Furthermore, a separate experiment was conducted to monitor a normally growing *E. coli* culture in LB medium. When the OD_600_ reached 4.1, acetic acid was introduced at a final concentration of 5 mm. A sharp reduction in OD_600_ from 4.1 to 1.4 was recorded within just 1.5 h, when the pH of the fermentation broth was less than 4.4, indicating severe cell lysis or death.

**FIGURE 4:**
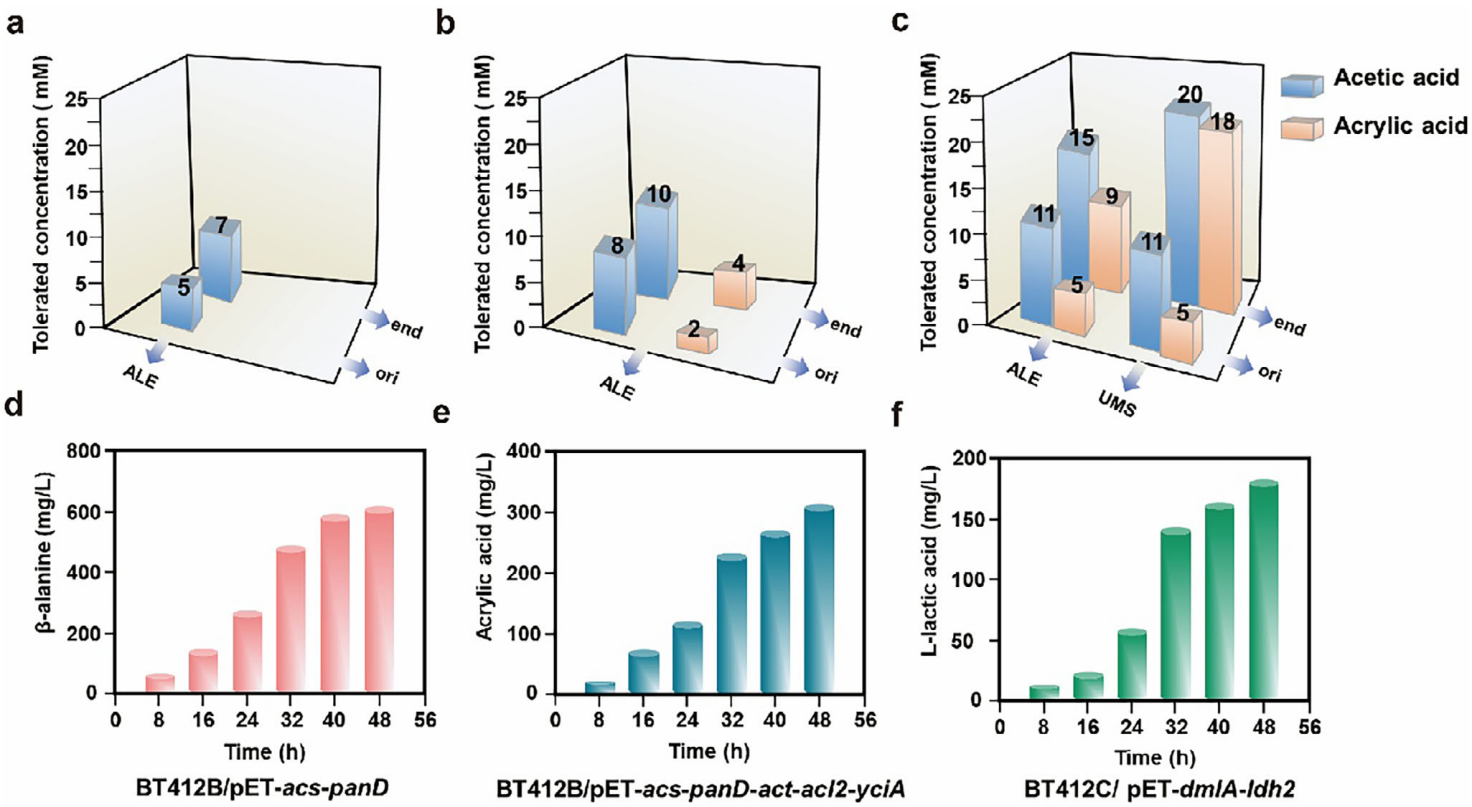
Adaptive evolution of strains under different conditions. (a,b) Adaptive evolution of strains under different conditions. (a) The XKX103 strain was adapted through 180 serial subcultures (every 12 h, 2% v/v transfer). Acetic acid was increased by 1 mm when OD_600_ reached ≥4.0. (b) The BT180 strain was adapted through 60 serial subcultures (every 12 h, 2% v/v transfer). Both acetic acid and acrylic acid concentrations were increased by 1 mm when OD_600_ reached ≥4.0. c. The BT240 strain, in which *polB* gene was overexpressed, was adapted through 172 serial subcultures (every 12 h, 2% v/v transfer), with both acetic acid and acrylic acid concentrations increased by 1 mm when OD_600_ reached ≥4. The BT240 strain without *polB* overexpression served as the control. d‐f. Fermentations for β‐alanine, acrylic acid, and L‐lactic acid production were conducted using the acetic acid and acrylate tolerant strain BT412‐derived strains BT412B/pET‐*acs*‐*panD* (*panD*: RBS15000 sequence) BT412B/pET‐*acs*‐*panD*‐*act‐acl2*‐*yciA* (*panD*: RBS15000 sequence, *yciA*: RBS8000 sequence), BT412C/pET‐dmlA^*^‐ldh2 (*ldh2*: RBS20000 sequence), respectively, in 2L shake flasks. The initial acetic acid concentration was set at 20 mm, and the fermentations were carried out at 37°C with an agitation speed of 220 RPM for 48 h. Samples were taken every 8 h for analysis. Reactions above were performed in triplicate (n = 3 biologically independent samples) and data are presented as mean values ± SD.

Adaptive laboratory evolution (ALE) was employed to address this particular problem. Consequently, ALE was performed on the parental BL21 strain. Following 180 serial passages, 7 mm acetic acid‐tolerant BT180 strain was obtained (Figure 4a). Furthermore, it was noted that *E. coli* growth was considerably inhibited when acrylic acid concentrations reached 2 mm, *E. coli* growth was significantly suppressed. The ALE strategy was employed to acclimatize the BT180 strain to acrylic acid and acetic acid. After 60 passages, 4 mm acrylic acid‐ and 8 mm acetic acid‐tolerant BT240 strain was obtained (Figure 4b). However, these strains were still not suitable for subsequent large‐scale fermentation due to limited improvement in acetic acid and acrylic acid tolerance. Thus, rapid screening of enhanced *E. coli* strains with acetic acid‐ and acrylic acid‐tolerant was considered crucial in this study. The tolerance of strains could be rapidly improved by impairing the genetic stability of genomic information through modulating physiological processes [34]. For instance, Hisaji Maki et al. achieved an increase of 980 times in the spontaneous mutation rate of *E. coli* by knocking out the *MutM* and *MutY* genes associated with oxidative damage [35]. Other studies obtained strains with gradient mutation rates by overexpressing the *dinB* gene to meet specific mutation rate requirements for complex phenotypes [36, 37, 38]. It was reported by multiple articles that the intracellular level of the *polB* gene was directly correlated with the cellular spontaneous mutation rate [39, 40, 41, 42]. However, this mechanism had not been applied to strain domestication in related studies.

**FIGURE 5:**
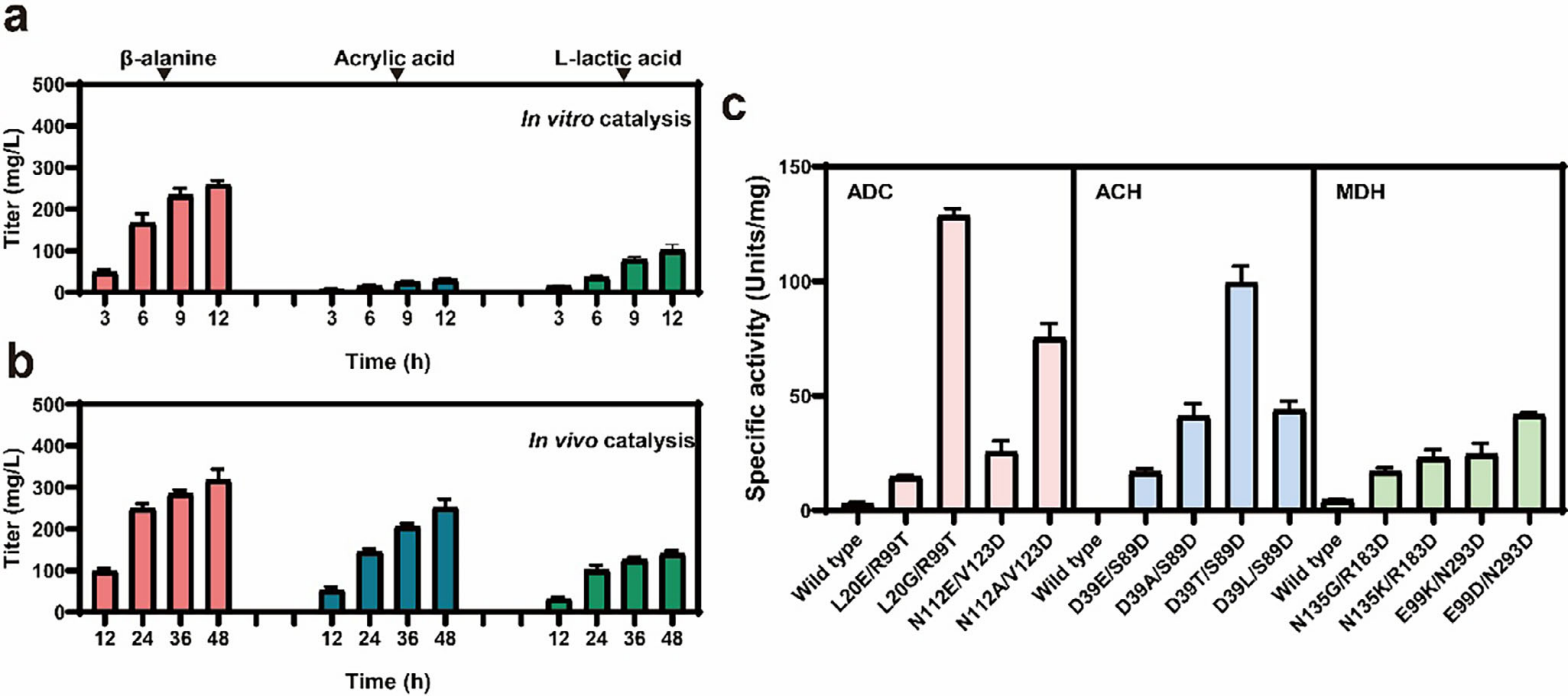
In vitro catalysis and shake flask fermentation of C3 compounds.

In this study, an inducible promoter was chosen to control the *polB* gene encoding DNA polymerase II, aiming to amplify *E. coli* response to DNA damage and its spontaneous genomic mutation rate. This was also the first time that overexpression of the *polB* gene on a plasmid had been applied to the evolution of *E. coli*. We constructed plasmids with 10 different RBS sequences combined with the *polB* gene for expression. After screening positive clones, BT240 strain with a spontaneous genomic mutation rate increased by 38.5 times were obtained (Table S3 and Table S4). After 172 passages, 18 mm acrylic acid‐ and 20 mm acetic acid‐tolerant BT412 strain (Table S4) was obtained. To prevent further unfavorable mutations in BT412 strain, the plasmid expressing the *polB* gene was removed. In order to further improve acrylic acid production, two endogenous pathways were truncated. These were the fumarate consumption pathway controlled by fumarate dehydrogenase expressed by the *fumABC* gene, and the acrylyl‐CoA consumption pathway controlled by acrylyl‐CoA reductase expressed by the *acuI* gene [43]. Finally, BT412B/pET‐*acs*‐*panD*‐*act‐acl2*‐*yciA* (*panD*: RBS15000 sequence, *yciA*: RBS8000 sequence) strain produced 252.4 mg/L of acrylic acid (Figure 5), with a yield of 0.84 g/g acetic acid, which represented the highest titer of acrylic acid as acknowledged [43]. Furthermore, the CO_2_‐derived acetic acid showed great advantages as a fermentation feedstock compared with petroleum‐based acetic acid (Supporting Information Methods), which was consistent with previously reported literature [12].

For the in vitro enzymatic assay: acetic acid was used as the substrate at 5 mm and incubated for 12 h in a reaction volume of 250 µL with shaking at 150 RPM and a temperature of 32°C. For the microbial shake‐flask fermentation: acetic acid was added at 5 mm concentration and fermented for 48 h in a volume of 400 mL. The inducer IPTG was added at 4 h to a final concentration of 0.4 mm with shaking at 300 RPM and a temperature of 37°C. Reactions above were performed in triplicate (n = 3 biologically independent samples) and data are presented as mean values ± SD.

A strategy was pioneered through the deliberate disruption of DNA damage repair systems, which significantly enhanced microbial tolerance to acrylic acid [44]. This approach eliminated the stochasticity and reverse mutation risks inherent in conventional mutagenesis methods, while enabling rapid accumulation of beneficial mutations to support high‐concentration substrate fermentation [45, 46]. Shao et al. reported an orthogonal transcription mutation system, the system demonstrated high specificity and minimal off‐target effects [47]. However, it required complex plasmid construction and optimization, and did not facilitate whole‐cell adaptation to environmental stresses. Diercks et al. introduced a powerful exogenous replisome into *E. coli*, significantly increasing the mutation rate [48]. However, it may impose an unknown burden on the host's physiology and potentially lead to system instability due to plasmid incompatibility or toxicity. In future studies, we will also construct a series of strains with different mutation rates to expand this mutant strain library, so as to meet the varying tolerance requirements of different experiments. Currently, acrylic acid production is heavily dependent on petroleum‐based feedstocks [49, 50, 51, 52]. Ko, Yoo‐Sung et al. engineered a novel biosynthetic pathway for acrylic acid based on β‐alanine. They use a delayed induction method to mitigate the toxic effects of acrylic acid on cells [43]. However, this strategy merely postponed the onset of toxicity rather than substantively alleviating it. Furthermore, the impact of acetic acid in the system was not addressed, despite its known inhibitory effects on microbial metabolism. By employing *polB* overexpression to accelerate bacterial evolution, we developed a robust strain capable of tolerating high concentrations of acrylic acid and acetic acid. This strategy fundamentally addresses the issue of growth inhibition caused by product toxicity and acetic acid during fermentation.

### 2.4 Biosynthesis of β‐Alanine in Engineered Strains

Based on the multi‐enzyme catalysis results in the in vitro experiments above, the engineered *E. coli* strains XKX103/ pET‐*acs*‐*panD* (*panD*: with RBS10000 sequence) (Table S4) was constructed to examine its β‐alanine production performance. XKX103/ pET‐*acs*‐*panD* (*panD*: with RBS10000 sequence) strain produced 48.36 mg/L of β‐alanine, with a yield of 0.16 g/g acetic acid. In addition, the concentration of acetic acid in the fermentation broth was measured to be 11.64 mg/L. These results suggested interference from competing endogenous pathways within the *E. coli* host for the constructed pathway, the competitive relationship between destined chemicals synthesis and byproducts production routes. It was noted that fumarate was partially diverted toward β‐alanine synthesis and partially converted to malate [53], where it re‐entered TCA cycle. Consequently, we knocked out the *fumABC* gene (encoding fumarate dehydrogenase) to eliminate by‐product synthesis pathway (Figure 3b). Then, strain XKX114/ pET‐*acs*‐*panD* (*panD*: with RBS10000 sequence) strain produced 259.25 mg/L of β‐alanine using 5 mm acetic acid as the substrate in a 2 L fermentation system, with a yield of 0.86 g/g acetic acid, higher than the literature value of 0.53 g/g [54].

β‐alanine, as a high‐value chemical, conventional chemical synthesis suffers from toxic reagents and high energy consumption, while biological methods face limitations in substrate cost and enzyme stability [54, 55, 56]. To further enhance β‐alanine production, first, precursor supply and carbon flux redirection were intensified through overexpression of phosphoenolpyruvate carboxylase or heterologous expression of pyruvate carboxylase from *Corynebacterium glutamicum*, which elevated oxalo acetic acid flux [57]. Concurrently, genes responsible for acetic acid synthesis and competing TCA cycle branches such as *fumABC* were deleted to minimize carbon loss. Carbon uptake was dynamically regulated using a pyruvate‐responsive system to prevent metabolic overflow. Second, cellular tolerance was augmented by supplementation of osmoprotectants, including glycine betaine to mitigate β‐alanine induced osmotic stress, and an β‐alanine‐responsive promoter system was employed to dynamically express tolerance genes such as *betT*, thereby improving cell viability under high productivity conditions [58]. Catalytic efficiency was enhanced through engineering of L‐aspartate‐α‐decarboxylase, exemplified by the K104S variant of ADC, which alleviated product inhibition [57]. This integrated approach, combining refined metabolic pathway reconstruction with dynamic tolerance engineering—demonstrated potential for efficient acetic acid‐based β‐alanine production, offering a novel methodology for converting CO_2_ into high‐value chemicals.

### 2.5 Biosynthesis of L‐Lactic Acid in Engineering Strains

A new pathway has been established for the stereospecific biosynthesis of L‐lactic acid, directly from CO_2_‐derived acetic acid. This approach achieves unprecedented chiral control in CO_2_ reduction, transforming a sustainable feedstock into a high‐purity bioproduct. The results above reported outline a successful multi‐enzyme catalysis in the in vitro experiments for L‐lactic acid, based on in vitro catalysis analysis, we modified the RBS sequence of the *ldh2* gene and subsequently cloned the relevant genes into *E. coli*, the engineered *E. coli* strains BL21/pET‐dmlA^*^‐ldh2 (*ldh2*: RBS2000 sequence) (Table S4) was constructed to examine its L‐lactic acid production performance. However, we did not detect L‐lactic acid in the fermentation samples. As verified from the literature, within the constructed L‐lactic acid synthesis pathway, a competing endogenous pathway was existed in *E. coli* [59].

Following knockout of *ldhA* gene (Figure 3b), strain BT412C was constructed (Table S4). Upon introduction of plasmid pET‐dmlA^*^‐ldh2 (*ldh2*: RBS2000 sequence), shake‐flask fermentation was conducted using 5 mm acetic acid as the substrate, producing 27.4 mg/L L‐lactic acid. SDS‐PAGE analysis revealed that expression of *ldhA*‐encoded L‐lactate dehydrogenase was substantially low, suggesting a bottleneck by L‐lactate dehydrogenase in the terminal conversion step. To address this limitation, five distinct ribosomal binding site variants were designed to optimize enzyme expression. Plasmid pET‐dmlA^*^‐ldh2 (*ldh2*: RBS20000 sequence) incorporating the optimized RBS was subsequently constructed and transformed into BT412C strain. This engineering strategy culminated in an L‐lactic acid titer of 141.24 mg/L under identical fermentation conditions. with a yield of 0.47 g/g acetic acid. Compared with those in other studies, the yields of the three chemicals had obvious advantages, as shown in Table S7.

L‐Lactic acid is widely used in food and beverage manufacturing [60, 61]. Currently, commercial‐scale lactic acid production is predominantly dependent on sugar‐derived feedstocks such as corn starch and sugarcane [62]. As an abundant carbon resource, CO_2_ can be converted to L‐lactic acid by acetic acid, Tong et al. produced 7.83 mg/L of L‐lactic acid via 95 h in vitro multi‐enzyme catalysis with CO_2_ [63], there was still a lot of room for improvement. Direct utilization of CO_2_ as the feedstock was implemented, reducing process steps by 33% [64]. The approach we provided shows a more streamlined and scalable technical option for carbon‐negative biomanufacturing.

### 2.6 Fed‐Batch Fermentation of C3 Compounds and Economic Benefit Analysis

**FIGURE 6:**
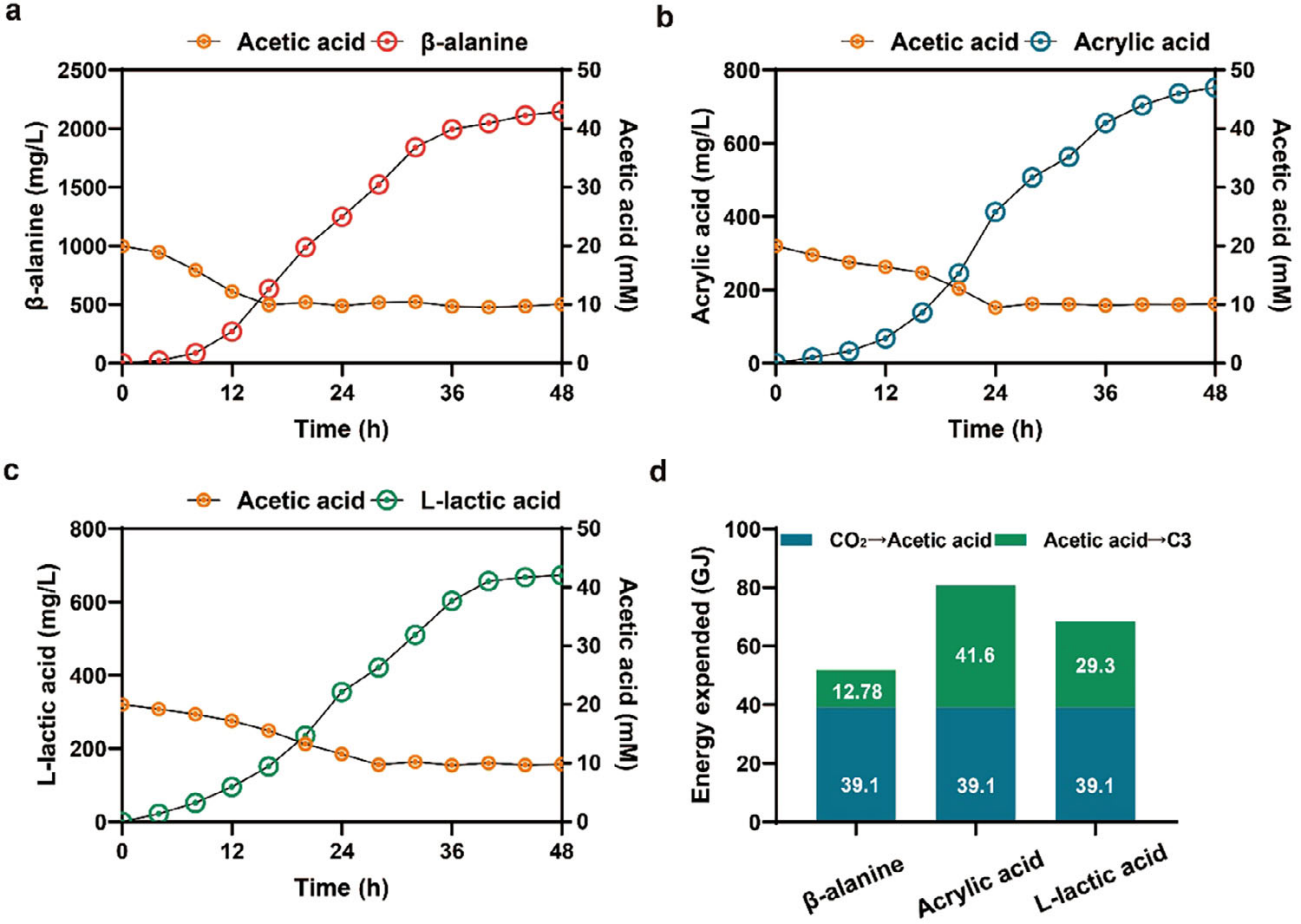
Fed‐batch fermentation and economic benefit accounting of C3 compounds. (a–c) Fed‐batch fermentation with three C3 compounds: acetic acid was initially supplied a concentration at 20 mm, feeding was initiated when concentrations fell below 10 mm and subsequently maintained at 10 mm until completion. The 48 h fermentation was conducted at 37°C with 30% dissolved oxygen (DO), where agitation was controlled to maintain DO. Samples were collected every 4 h and averaged from triplicate measurements. (d) Energy calculations and economic assessments for both the CO_2_ to acetic acid conversion and the acetic acid to C3 chemicals processes were performed, with algorithms detailed in Method S1.

The fed‐batch fermentation experiments were conducted in a 5 L fermenter. An initial acetic acid concentration of 20 mm was supplied in the fermentation broth, with subsequent acetic acid concentration maintained at 10 mm. For β‐alanine fermentation, BT412A/pET‐*acs*‐*panD* (*panD*: with RBS10000 sequence) strain was used. As shown in Figure 6a–c, a final β‐alanine concentration of 2,147.1 mg/L was achieved at 48 h, with an overall productivity of 44.7 mg/L/h and a yield of 0.72 g/g acetic acid. For acrylic acid fermentation, BT412B/pET‐*acs*‐*panD*‐*act‐acl2*‐*yciA* (*panD*: RBS15000 sequence, *yciA*: RBS8000 sequence) strain was used. A final acrylic acid concentration of 752.4 mg/L was attained, demonstrating an overall productivity of 15.7 mg/L/h and a yield of 0.54 g/g acetic acid, For L‐lactic acid fermentation, BT412C/pET‐dmlA^*^‐ldh2 (*ldh2*: RBS20000 sequence) strain was used. A final L‐lactic acid concentration of 672.9 mg/L was obtained, resulting in an overall productivity of 7.8 mg/L/h and a yield of 0.68 g/g acetic acid, higher than the literature value [11, 12, 13, 16, 65], The purity of acrylic acid, β‐alanine, and L‐lactic acid in the fermentation broth reached 92.4%, 84.2%, and 81.8%, respectively, as shown in Table S6. These results confirmed that the engineered stains BT412A/pET‐*acs*‐*panD* (*panD*: with RBS10000 sequence), BT412B/pET‐*acs*‐*panD*‐*act‐acl2*‐*yciA* (*panD*: RBS15000 sequence, *yciA*: RBS8000 sequence), and BT412C/pET‐dmlA^*^‐ldh2 (*ldh2*: RBS20000 sequence) were capable of high level β‐alanine/acrylic acid/L‐lactic acid production, with productivity and yield.

To further evaluate the industrial application prospects of the engineered strain, cost accounts was conducted. As shown in Figure 6d and Method S1, this study demonstrates that converting 1 ton of CO_2_ to acetic acid requires 39.1 GJ of energy, predominantly consumed in the electrocatalytic module. The energy demands for synthesizing C3 chemicals from acetic acid are quantified as follows: 12.78 GJ/t for β‐alanine, 29.3 GJ/t for acrylic acid, and 41.6 GJ/t for L‐lactic acid, and the calculation method is described in Method S1. This study demonstrates a groundbreaking advancement in low‐energy electrocatalytic conversion of CO_2_ into C3 chemicals. As shown in Table S11, the production routes for β‐alanine, L‐lactic acid, and acrylic acid presented in this study with an estimated production cost of $1476, $2481, and $2243 per ton, respectively. Compared to traditional methods [43, 66, 67], our approach incurred a higher energy cost due to the limitation on equipment specifications. However, our microbial electroreduction system achieved carbon capture, utilization, and storage directly without additional processing, and then gain more economic benefits. In future research, reductions in energy consumption could be achieved by optimizing fermentation processes, while metabolic engineering could improve the production and yield of β‐alanine, L‐lactic acid, and acrylic acid, ultimately lowing overall costs. Combining revolutionary carbon conversion efficiency with strong commercial potential, this work provides an innovative pathway for zero‐carbon chemicals production.

## 3 Conclusion

We successfully developed a tandem microbial electroreduction‐biosynthesis platform for the efficient conversion of CO_2_ into valuable C3 chemicals. By utilizing a Co‐P alloy‐coated stainless‐steel mesh working electrode, enhanced acetic acid production of 0.34 ± 0.01 g L^−1^ day^−1^ was achieved. Through synthetic biology approaches, we achieved the targeted biosynthesis of acrylic acid, L‐lactic acid, and β‐alanine from CO_2_‐derived acetic acid. Furthermore, the implementation of a novel ultrahigh mutation system (UMS) improved microbial acetic acid tolerance, facilitating robust bioproduction. This work demonstrates the feasibility of using CO_2_ as a carbon source for biomanufacturing, expands the product diversity of microbial electrosynthesis systems, and provides a sustainable alternative to conventional petroleum‐derived chemical production routes. In future studies, we aim to develop a continuous microbial electroreduction and biosynthesis coupling system that harmonizes the reaction rates between the microbial electroreduction module and the biosynthesis module, which could further amplify the advantages of the microbial electroreduction module developed in our system with high‐purity acetate production in the anode pool.

## 4 Methods

### 4.1 Cloning and Purification of Enzymes

The detailed information of the enzymes used in this experiment is presented in Table S2, including their abbreviations, full names, source organism, and the Gibbs free energy changes associated with the reactions they catalyze. The information on the strains used in this article is shown in Table S4, and information on the enzymes required to produce a specific C3 compound is detailed in Table S5.

For in vitro catalytic experiments, the requisite heterologous genes were co‐expressed on the pET28a plasmid, which was governed by the lac operon and T7 promoter, or individually expressed on the pET28a plasmid. *E. coli* MG1655 and *B. subtilis 168* were maintained as laboratory stock strains. Unless otherwise indicated, all enzyme‐encoding plasmids were introduced into *E. coli* BL21(DE3) and expressed in LB medium containing 50 µg/mL kanamycin and 100 µg/mL ampicillin. Four hundred milliliters of culture was inoculated with 4 mL of an overnight‐cultured seed medium in the same medium and grown to an OD_600_ of 0.6–0.8 at 37°C. The cultures were then induced with 0.8 mm IPTG and allowed to express the recombinant enzymes at 16°C for 20 h.

Cells were harvested by centrifugation at 4000 RPM and resuspended in 20 mL of binding buffer (20 mm NaH_2_PO4, 500 mm NaCl, and 30 mm imidazole, pH 7.6). Cells were lysed using a high‐pressure cell disruptor. Unbroken cells and debris were removed by centrifugation at 10 000 RPM for 1 h. The clarified lysate was incubated with Ni‐NTA resin for 1 h at 4°C. The resin was washed three times with 5 volumes of wash buffer (20 mm NaH_2_PO_4_, 500 mm NaCl, and 100 mm imidazole, pH 7.6). Enzymes were eluted with 5 mL of elution buffer (20 mm NaH_2_PO_4_, 500 mm NaCl, and 500 mm imidazole, pH 7.6).

The eluted enzyme buffer was exchanged into enzyme storage buffer (50 mm Tris‐HCl, pH 8.0) using ultrafiltration, and 20% glycerol was added as a cryoprotectant. Aliquoted samples were stored at −80°C, and glycerol was removed by ultrafiltration prior to use. Enzyme concentrations were determined using the Bradford assay, and the enzyme gel is shown in Figure S1.

### 4.2 Cultivation Conditions

In the context of plasmid construction and routine experimental procedures, *E. coli* strains, either wild‐type or genetically modified, were commonly cultivated on LB medium. Where necessary, antibiotics were incorporated into the medium to ensure selection of the desired strains.

For *S. ovata* cultivation in bio‐electrocatalytic acetic acid production, prepare three solutions: (1) Basal medium (5 L): 4.59 g NaCl, 1.74 g K_2_HPO_4_, 1.14 g NH_4_Cl, 10.73 g Na_2_HPO_4_·7H_2_O, 4.69 g NaH_2_PO_4_·H_2_O, 2.5 g MgSO_4_·7H_2_O, 1.25 g CaCl_2_·2H_2_O, 0.01 g FeSO_4_·7H_2_O, 0.001 g NaHSeO_3_, adjusted to 5 L with deionized water; (2) Trace element solution (1 L, pH 3.4): 0.1 g ZnSO_4_·7H_2_O, 0.03 g MnCl_2_·4H_2_O, 0.3 g H_3_BO_3_, 0.2 g CoCl_2_·6H_2_O, 0.01 g CuCl_2_·2H_2_O, 0.02 g NiCl_2_·6H_2_O, 0.03 g Na_2_MoO_4_·2H_2_O; (3) Vitamin mixture (1 L): 2 mg biotin, 2 mg folic acid, 10 mg pyridoxine‐HCl, 5 mg each of thiamine‐HCl·2H_2_O, riboflavin, nicotinic acid, D‐Ca‐pantothenate, p‐aminobenzoic acid, lipoic acid, and 0.1 mg vitamin B_12_. Combine 250 mL basal medium with 0.5 mL trace elements and vitamins. After cooling, add 1 g NaHCO_3_ and 0.5 g yeast extract, then sparge with 80% N_2_/20% CO_2_ for 10 min. Aliquot 15 mL into anaerobic vials, sparge with 20% CO_2_/80% H_2_ for 1 min, seal, and inoculate with 0.5 mL sterile 10% betaine and 0.4 mL reducing solution (1.5 g L‐cysteine‐HCl and 1.5 g Na_2_S·9H_2_O in 100 mL deionized water, filtered and sparged). Incubate *S. ovata* at 30°C under 3 atm H_2_; colonies emerge within 24 h on heterotrophic medium [15].

For engineered *E. coli* (acrylic acid production), streak on LB agar with 50 µg/mL kanamycin, incubate at 37°C for 12–16 h, then inoculate single colonies into 50 mL LB medium (37°C, 200 RPM, 12–16 h). Transfer 1% seed culture to 400 mL selective medium (composition in supplement), induce with IPTG and bio‐electrocatalysis filtrate at OD_600_ = 0.6‐0.8, and ferment at 37°C, 220 RPM.

### 4.3 Electrochemical Acetic Acid Production Module

This study employed a customized scale‐up H‐cell with a total volume of 2 L (each chamber of 1 L) as the electrochemical reactor (Figure 1). The cathodic and anodic chambers of the H‐cell were ionically connected by an AEM (FAA‐3‐PK‐75). Stainless steel mesh loaded with Co‐P catalyst (5 cm × 5 cm), Ag/AgCl (1 m KCl, CH Instruments) and Pt mesh (1 cm × 2 cm) were used as the working electrode, reference electrode, and counter electrode, respectively. To maintain a near‐neutral pH environment for sustainable operation of *S. ovata* during electrochemical measurements, a modified autotrophic DSMZ 311 medium (15×phosphate concentration, initial pH adjusted to 6.7, Table S1) was used as the electrolyte. A volume of 700 mL of electrolyte was introduced into the cathodic and anodic chambers of the H‐cell, respectively. Before *S. ovata* inoculation, the system was first operated continuously for 24 h under galvanostatic mode at 5 mA. During this abiotic phase, continuous bubbling of a mixture gas (80% N_2_/20% CO_2_) removed residual O_2_ in the cathodic chamber, establishing a completely anaerobic gaseous environment for *S. ovata*. Subsequently, sterilized perfluorocarbon nanoemulsions (5% v/v) and hydrogen‐grown *S. ovata* cells (7% v/v, corresponding to an OD_545_ of 0.38) were inoculated into the cathodic chamber. After bacterial inoculation, a constant current of 10 mA was applied for 24 h to provide a mild electrochemical environment for bacterial acclimation. The integrated system eliminates intermediate steps, such as purification, separation, and pH adjustment, required in conventional abiotic approaches. The acetate‐containing electrolyte can be directly concentrated and utilized in downstream biosynthesis, enabling streamlined intermediate recovery. Furthermore, the 5 mA (24 h) and 10 mA (24 h) stages represent a one‐time electrochemical deoxygenation and microbial acclimation procedure, which was common and necessary in anaerobic MES systems. Importantly, *S. ovata* was not discarded at the end of each operation but can be maintained and reused during continuous or long‐term operation. Therefore, this initial 48 h period system preparation does not constitute a fundamental increase in system complexity. Next, the system was run at 50 mA (2.0 mA cm^−2^) for 5 days to produce acetic acid. Starting at 0 h, aliquots of the medium were sampled every 24 h from both cathodic and anodic chambers for analysis of cell density, pH, and acetic acid concentration. The flow rate of the cathodic gas mixture (80% N_2_/20% CO_2_) was maintained at 3 mL/min throughout the experiments. All the electrochemical characterizations were performed using CORRTEST CS350M potentiostat (Wuhan CorrTest Instruments Co., Ltd).

### 4.4 Measurement of Hydrogen Generation Rate

The H_2_ generation rate (0.93 mmol h^−^^1^) was a theoretical estimate based on Faraday's law, not a direct experimental measurement. This calculation assumes: (i) the Co‐P catalyst‐loaded stainless‐steel mesh electrodes exhibit near‐unity Faradaic efficiency for the hydrogen evolution reaction (FEH_2_ ≈ 100%), (ii) complete electrochemical deoxygenation prior to inoculation, and (iii) the absence of detectable byproducts other than H_2_ during the deoxygenation stage. Consequently, the entire 50 mA current was attributed to HER for this estimate, which was used only for mechanistic discussion and order‐of‐magnitude analysis, not for quantitative energy efficiency calculations.

### 4.5 Purification of Acetic Acid

Acetic acid was purified from module I (Figure S5). First, the acetate‐containing electrolyte was centrifuged (8000 RPM, 10 min) and filtered to remove biological cells and insoluble substances. Subsequently, the electrolyte was concentrated to 20% of its initial volume by solvent evaporation at 120°C. The pH was then acidified to 2.0 using concentrated sulfuric acid to convert acetate to acetic acid.

### 4.6 Enzymes Activity Assay

The activity of ADC was determined by evaluating the conversion of aspartate to β‐alanine using HPLC. The reaction mixture contained 1 mm aspartate and 1 µm ADC purified enzyme in a final volume of 100 µL. After incubation at 37°C for 1 h, 40 µL of the reaction mixture was immediately mixed with an equal volume of 3% perchloric acid in water, which contained 393 µm δ‐amino‐n‐valeric acid as an internal standard. Precipitated proteins were removed by centrifugation at 10,000 RPM, and 50 µL of the supernatant was then added to 100 µL of sodium borate buffer (0.4 m, pH 9.5). The samples were stored at 4°C until HPLC analysis.

The activity of ACT, ACAL, and ACH were determined by evaluating the conversion of β‐alanine to acrylic acid using HPLC. Purified enzymes (ACT, ACAL, and ACH) were mixed at an equivalent molar ratio (1 µm ACT, 1 µm ACAL and 1 µm ACH), and incubated at 32°C for 1 h while 5 mm β‐alanine and 1 mm acetyl‐CoA were supplemented as substrates. The total 250 µL of assay mixture was incubated at 32°C for 1 h, followed by boil at 100°C for 10 min. Precipitated proteins were removed by centrifugation at 10,000 RPM, and the samples were stored at 4°C until HPLC analysis.

The activity of MDH determined by evaluating the conversion of malate to pyruvate using HPLC. The reaction mixture contained 1 mm malate 1 µm MDH, and 1 mm NAD^+^ in a final volume of 100 µL. And incubated at 37°C for 15 min, using Beyotime assay kit to monitor changes in NADH levels, then to determine MDH activity.

### 4.7 Chemicals Production from Acetic Acid in vitro

The primary acrylic acid in vitro catalytic synthesis module consists of 1 µm ACS, 1 µm CS, 1 µm AH, 1 µm ICL, 1 µm SDH, 1 µm AspA, 1 µm ADC, 1 µm ACT, 1 µm ACAL, 1 µm ACH, 1 mm acetyl‐CoA, 5 mm MgCl_2_, 50 mm KCl, and 50 mm NaCl in a 100 mm Tris‐HCl buffer (pH 7.5). To this, 5 mm acetic acid was added as the substrate for the in vitro catalytic reaction, along with a crude extract derived from the cell culture supernatant of the engineered *E. coli* strain corresponding to an optical density (OD_600_). A 10 kDa molecular weight cut‐off (MWCO) Millipore ultrafiltration column was employed to wash the lysate with 50 mm Tris‐HCl (pH 8.0) twice, followed by adjustment to the desired volume to obtain a crude cell mixture. The resultant 250 µL reaction mixture was incubated at 32°C for 12 h, after which the system was heat‐inactivated by sealing and exposure to 100°C for 10 min. Quantification of acrylic acid within the reaction system was performed using high‐performance liquid chromatography (HPLC), with further analysis conducted via HPLC‐MS for the detection of acrylic acid within the system.

The primary L‐lactic acid in vitro catalytic synthesis consists of 1 µm ACS, 1 µm CS, 1 µm CitB, 1 µm ICL, 1 µm SDH, 1 µm MDH, 1 µm LLDH, 5 mm MgCl_2_, 50 mm KCl, and 50 mm NaCl in a 100 mm Tris‐HCl buffer (pH 7.5). To this, 5 mm acetic acid was added as the substrate for the in vitro catalytic reaction, along with a crude extract derived from the cell culture supernatant of the engineered *E. coli* strain corresponding to an optical density (OD_600_). A 10 kDa molecular weight cut‐off (MWCO) Millipore ultrafiltration column was employed to wash the lysate with 50 mm Tris‐HCl (pH 8.0) twice, followed by adjustment to the desired volume to obtain a crude cell mixture. The resultant 250 µL reaction mixture was incubated at 32°C for 12 h, after which the system was heat‐inactivated by sealing and exposure to 100°C for 10 min. Quantification of L‐lactic acid within the reaction system was performed using HPLC, with further analysis conducted via HPLC‐MS for the detection of LA within the system.

The primary β‐alanine in vitro catalytic synthesis consists of 1 µm ACS, 1 µm CS, 1 µm CitB, 1 µm ICL, 1 µm SDH, 1 µm AspA, 1 µm ADC, 5 mm MgCl2, 50 mm KCl, and 50 mm NaCl in a 100 mm Tris‐HCl buffer (pH 7.5). A 10 kDa molecular weight cut‐off (MWCO) Millipore ultrafiltration column was employed to wash the lysate with 50 mm Tris‐HCl (pH 8.0) twice, followed by adjustment to the desired volume to obtain a crude cell mixture. The resultant 250 µL reaction mixture was incubated at 32°C for 12 h, after which the system was heat‐inactivated by sealing and exposure to 100°C for 10 min. Quantification of β‐alanine within the reaction system was performed using HPLC, with further analysis conducted via HPLC‐MS for the detection of β‐alanine within the system.

### 4.8 Flask‐Shaking Fermentation

To determine the titers of C3 chemicals produced by our engineered strains using acetic acid, flask fermentation was conducted in this study. The strains were pre‐cultured in LB medium, and cultures grown in tubes for 12–16 h were inoculated into fermentation medium at a 2% (v/v) ratio. Fermentation was carried out with 5 mm acetic acid as the substrate for 48 h under conditions of 37°C and 300 RPM. After fermentation, 1 mL samples were collected and centrifuged at 10 000 RPM, followed by filtration of the supernatant for HPLC analysis. The fermentation process using industrially sourced acetic acid was identical to that using CO_2_‐derived acetic acid.

### 4.9 Fed‐Batch Fermentation

To further explore the process conditions for scale‐up cultivation, we performed fed‐batch fermentation using the engineered strain with acetic acid as the substrate in a 5 L bioreactor with a working volume of 2 L. The seed culture was prepared in LB medium. The initial acetic acid concentration was set at 20 mm, and the dissolved oxygen (DO) level was maintained at 30%. The agitation speed was coupled with DO and adjusted in real‐time based on fermentation performance. When the acetic acid concentration decreased to 10 mm, a feeding strategy was initiated to maintain it at this level. The total fermentation time was 48 h. After fermentation, samples were collected, centrifuged at 10 000 RPM, and the supernatants were filtered and subjected to HPLC analysis.

### 4.10 Analytic Methods

The cellular density was measured using a UV–vis spectrophotometer at an absorbance of 600 nm (OD_600_). The cellular fermentation broth was subjected to centrifugation at 10 000 RPM for a duration of 30 min. The resulting supernatant was then filtered through a 0.22 µm membrane filter. Acetic acid detection was carried out by high‐performance liquid chromatography (HPLC) equipped with a MetaCarb 87H column (7.8 mm × 300 mm, Agilent Technologies, Santa Clara, CA), with the column oven set at a temperature of 25°C and utilizing 5 mm sulfuric acid as the mobile phase at a flow rate of 0.5 mL/min.

The quantification of β‐alanine was performed using the o‐phthalaldehyde derivatization method, with separation achieved on a ZoRBAX Eclipse AAA column (5 µm, 3.0 mm × 150 mm, Agilent Technologies).

The concentration of fumaric acid was determined by HPLC employing a ShimNex CS C18 column (5 µm, 4.6 mm × 250 mm, Shimadzu), maintained at a column temperature of 35°C. The mobile phase consisted of solvent A, 0.2% (w/v) phosphoric acid (80 wt.%), and solvent B, acetonitrile. The flow rate was set at 1.0 mL/min, the solution ratio remains the same. Fumaric acid was tested in our laboratory using a high‐phase liquid phase of LC‐20AD.

The concentration of acrylic acid was determined by HPLC employing a C18 plus column (5 µm, 4.6 mm × 150 mm, Agilent Technologies), maintained at a column temperature of 25°C. The mobile phase consisted of solvent A, 0.1% (w/v) phosphoric acid (85 wt.%), and solvent B, acetonitrile. The flow rate was set at 0.8 mL/min, with a gradient elution program as follows: a linear gradient from 0% to 70% B over 0 to 14 min; a subsequent linear gradient from 70% to 100% B between 14 and 14.5 min; and isocratic elution with 100% B from 14.5 to 15 min. Authentic standards of acrylic acid were sourced from McClean Platform. The HPLC of the C3 chemicals is shown in Figure S3. A subset of the experimental procedures was conducted at the Analytical and Testing Center of Chongqing University.

The concentration of pyruvate was determined by HPLC employing a shim‐pack SCR‐101H column (10 µm, 7.9 mm × 300 mm), maintained at a column temperature of 20°C. The mobile phase was used as a perchloric acid aqueous solution with a pH of 3.8, flowing at a rate of 0.8 mL/min. The experiment was completed in this laboratory.

The concentration of L‐lactic acid was determined by HPLC, the revised HPLC method is as follows: Column: CRS10 W (4.6 mm × 50 mm, 3 µm); Mobile phase: 0.1 mol/L CuSO_4_·5H_2_O aqueous solution, filtered through 0.45 µm membrane; Flow rate: 0.5 mL/min; Column temperature: 25°C; UV detection at 254 nm; Injection volume: 10 µL.

All plotted data in the figure and Supporting Information figure were analyzed using GraphPad Prism (version 8) or Origin (version 8). The chemical structure was drawn using ChemDraw (version 21.0). Multipaned figures were arranged using Adobe Illustrator 2020.

## Author Contributions

K.X., D.W., S.L., X.Z., and Y.S. conceived and designed the experiments. K.X., Y.K., X.Z., and Z.P. performed the experiments. D.W. and R.L. helped guide the pathway analyzing and gene editing. Z.P. helped complete the strain screening. K.X., J. Z., and D.W. analyzed the experimental data; K.X., Y.S., L. Z., and D.W. wrote the manuscript. All authors approved the final manuscript.

## Conflicts of Interest

The authors declare no conflicts of interest.

## Supporting information




**Supporting File**: advs73698‐sup‐0001‐SuppMat.docx.

## Data Availability

A reporting summary for this article is available as Supporting Information file. The main data supporting the findings of this study are available within the article and its Supporting Information figures. Figures 2, 4, 5 and 6 are provided as a Source Data file. Source data are provided with this paper. All the databases used in the study: MetaCyc: https://metacyc.org/, Uniprot database: https://www.uniprot.org/, eQuilibrator: https://equilibrator. weizmann.ac.il/, RBS Calculator: https://docs.denovodna.com/docs/rbs‐calculator, Source data are provided with this paper.
