# Supplementary material for: Combining Electrochemical Reduction with Biosynthesis for Directed Conversion of CO2 into a Library of C3 Chemicals
Source: Adv Sci (Weinh). 2026 Jan 4;13(17):e22097. doi: 10.1002/advs.202522097 (PMC13042692; doi:10.1002/advs.202522097)
Supplement: Supplementary file 1 — Supporting File: advs73698‐sup‐0001‐SuppMat.docx. [file ADVS-13-e22097-s001.docx]

**Supporting Information**

**Combining electrochemical reduction with biosynthesis for directed conversion of CO_2_ into a library of C3 chemicals**

Kaixing Xiao^1#^, Shanquan Liang^1#^, Xujun Zhao^2#^, Zhiyao Peng^1^, Ruoshi Luo^1^, Jikai Zong^1^, Ling Zhou^1^, Yude Su^2^*, Dan Wang^1^*

^1^Department of Chemical Engineering, School of Chemistry and Chemical Engineering, Chongqing University, Chongqing, 401331, P. R. China.

^2^Suzhou Institute for Advanced Research, University of Science and Technology of China, Suzhou, Jiangsu 215123, China.

***Corresponding author: Yude Su**

E-mail: suyude@ustc.edu.cn.

E-mail address: Suzhou Institute for Advanced Research, University of Science and Technology of China, Suzhou, Jiangsu 215123, China.

***Corresponding author: Dan Wang**

E-mail: dwang@cqu.edu.cn.

E-mail address: 55 Daxuecheng South Road, Shapingba District, Department of Chemical Engineering, School of Chemistry and Chemical Engineering, Chongqing University, Chongqing, 401331, P. R. China.

**Supplementary Table 1. Chemical compositions of the phosphate-enhanced DSMZ 311 medium. In comparison with the original DSMZ 311 medium (Methods), the concentration of phosphate buffer is increased by 15 times from 3.7 mM to 55.5 mM.**

| **Chemical compositions** | **Usage** | **Unit** |
| --- | --- | --- |
| K_2_HPO_4_ | 5.22 | g |
| KH_2_PO_4_ | 3.42 | g |
| Na_2_HPO_4_·7H_2_O | 32.19 | g |
| NaH_2_PO_4_·H_2_O | 14.07 | g |
| NH_4_Cl | 2.50 | g |
| MgSO_4_·7H_2_O | 2.50 | g |
| CaCl_2_·2H_2_O | 1.25 | g |
| FeSO_4_·7H_2_O | 0.01 | g |
| NaHSeO_3_ | 0.001 | g |
| NaHCO_3_ | 20.00 | g |
| Vitamin solution | 50 | mL |
| Trace element solution SL-10 | 5 | mL |
| Distilled water | 5000 | mL |

**Supplementary Table 2. Information of enzymes used in the enzymatic platform.**

| Enzyme Abbr. | Enzyme | | | EC number | Source Organism | | | △G`^0^ (Kcal/mol) ^[*]^ |
| --- | --- | --- | --- | --- | --- | --- | --- | --- |
| ACS | Acetyl-coenzyme A synthetase | | | EC:6.2.1.1 | *Escherichia coli (strain K12)* | | | -8.2 ± 0.8 |
|  | acetate + ATP + CoA = acetyl-CoA + AMP + diphosphate | | | | | | | |
| CS | Citrate synthase | | | EC:2.3.3.16 | *Escherichia coli (strain K12)* | | | -9.1 ± 0.8 |
|  | acetyl-CoA + ADP + oxaloacetate + phosphate = ATP + citrate + CoA | | | | | | | |
| AH | Aconitate hydratase B | | | EC:4.2.1.3 | *Bacillus subtilis (strain 168)* | | | 7.6 ± 4.6 |
|  | citrate = D-threo-isocitrate | | | | | | | |
| ICL | Isocitrate lyase | | | EC:4.1.3.1 | *Escherichia coli (strain K12)* | | | 8.7 ± 5.2 |
|  | D-threo-isocitrate = glyoxylate + succinate | | | | | | | |
| SDH | Succinate dehydrogenase | | | EC:1.3.5.1 | *Escherichia coli (strain K12)* | | | -24.1 ± 7.0 |
|  | a quinone + succinate ⇋ a quinol + fumarate | | | | | | | |
| AspA | Aspartate ammonia-lyase | | | EC:4.3.1.1 | *Eschericia coli*  *(strain K12)* | | | 13.2 ± 0.9 |
|  | L-aspartate = fumarate + NH_4_^+^ | | | | | | | |
| ADC | Aspartate 1-decarboxylase | | | EC:4.1.1.11 | *Eschericia coli*  *(strain K12)* | | | -16.9 ± 11.7 |
|  | H^+^ + L-aspartate =β-alanine + CO_2_ | | | | | | | |
| ACT | β-alanine CoA-transferase | | | - | *Anaerotignum propionicum* | | | 4.6 ± 10.9 |
|  | Beta-alanine + Acetyl-CoA =β-alanyl-CoA + Acetate | | | | | | | |
| ACAL | β-alanyl-CoA: ammonia lyase 2 | | | EC: 4.3.1.6 | *Anaerotignum propionicum* | | | 9.9 ± 4.9 |
|  | beta-alanyl-CoA = Acrylyl-CoA + ammonium | | | | | | | |
| ACH | Acyl-CoA thioester hydrolase | | | EC: 3.1.2.20 | *Escherichia coli (strain K12)* | | | -29.9 ± 2.8 |
|  | Acrylyl-CoA+H_2_O= Acrylic acid + CoA | | | | | | | |
| FH | fumarate hydratase | | EC: 4.2.1.2 | | | *Escherichia coli (strain K12)* | -3.4 ± 0.5 | |
|  | H_2_O + Fumarate ⇌ Malate | | | | | | | |
| MDH | malate dehydrogenase | | EC: 1.1.1.83 | | | *Escherichia coli (strain K12)* | 12.1 ± 6.1 | |
|  | NAD^+^ + Malate ⇌ NADH + CO_2_ + Pyruvate | | | | | | | |
| LLDH | L-lactate dehydrogenase | EC: 1.1.1.27 | | | | *Escherichia coli (strain K12)* | -23.7 ± 0.9 | |
|  | Pyruvate + NADH + H^+^⇌ NAD^+^ + L-lactate | | | | | | | |
| DLDH | D-lactate dehydrogenase | EC: 1.1.1.28 | | | | *Escherichia coli (strain K12)* | -20.6 ± 4.1 | |
|  | pyruvate + NADH + H^+^ ⇌ NAD^+^ + D-lactate | | | | | | | |

^*^The standard Gibbs free energy change (ΔG'^0^) of enzymatic reaction at pH 7.5 and ionic strength of 0.25 M (http://equilibrator.weizmann.ac.il/).

**Supplementary Table 3. The Lac+ colonies of strains M03 containing different plasmids on LB plates and their calculated SIM rates**

| Plasmid | Lac^+^ colonies per 10^6^ cells ^[a]^ | | | | | SIM rate (Lac^+^ colonies per 10^6^ cells per day) ^[b]^ |
| --- | --- | --- | --- | --- | --- | --- |
|  | 2 d | 3 d | 4 d | 5 d | 6 d |  |
| p01 | 155.4±20.8 | 160±5 | 182±6 | 192±5 | 205±8.8 | 12.2 |
| p03 | 134.8±10.5 | 75±15 | 133±35 | 153±48 | 166±20 | 7.8 |
| p04 | 485±35 | 518±40 | 541±41 | 565±48 | 585±65 | 25 |
| p05 | 31.6±5.8 | 33.5±2.5 | 36.9±3.2 | 38.8±4.7 | 42±5.1 | 2.6 |
| p06 | 134.1±18.6 | 168.4±5 | 201.4±8 | 229±15 | 258.1±19 | 31 |
| p07 | 9.2±1.6 | 55±2.4 | 155±3.6 | 276±3.7 | 353.4±4.5 | 38.5 |
| p08 | 75.6±15 | 77±10 | 79±11 | 83±14 | 88±17 | 3.1 |
| p09 | 11±1.2 | 26±5 | 47±7.2 | 69±8.2 | 75±9.9 | 16.5 |
| p10 | 94.8±9.4 | 105±10.3 | 139±12.2 | 164±13.2 | 192±17.8 | 24.3 |

^a^ Mean value ± standard deviations of three independent experiments

^b^ SIM rate was calculated by (The value at Day 6- The value at Day 2)/4

**Supplementary Table 4. Information on the strains and plasmid used in this study.**

| Strains and plasmids | | Relevant characteristics | source |
| --- | --- | --- | --- |
|  | strains | |  |
| BL21(DE3) | *F^-^ ompT hsdSB (rB^-^ mB^-^) gal dcm*(DE3) | | Tsingke |
| XKX004 | BL21(DE3) *△fumA* | | This study |
| XKX005 | BL21(DE3) *△fumB* | | This study |
| XKX006 | BL21(DE3) *△fumC* | | This study |
| XKX010 | BL21(DE3) *△fumAfumB* | | This study |
| XKX011 | BL21(DE3) *△fumBfumC* | | This study |
| XKX012 | BL21(DE3) *△fumAfumBfumC* | | This study |
| XKX045 | BL21(DE3) *△acuI* | | This study |
| XKX103 | BL21(DE3) *△panD* | | This study |
| XKX104 | BL21(DE3) *△panD△fumA* | | This study |
| XKX105 | BL21(DE3) *△panD△fumB* | | This study |
| XKX106 | BL21(DE3) *△panD△fumC* | | This study |
| XKX107 | BL21(DE3) *△panD△fumAfumB* | | This study |
| XKX110 | BL21(DE3) *△panD△fumBfumC* | | This study |
| XKX114 | BL21(DE3) *△panD△fumAfumBfumC* | | This study |
| XKX115 | BL21(DE3) *△panD△fumA△acuI* | | This study |
| XKX116 | BL21(DE3) *△panDfumB△acuI* | | This study |
| XKX119 | BL21(DE3) *△panDfumC△acuI* | | This study |
| XKX127 | BL21(DE3) *△panD△fumA△fumB△fumC△acuI* | | This study |
| XKX149 | BL21(DE3) *△aspA* | | This study |
| XKX150 | BL21(DE3) *△ldhA* | | This study |
| XKX152 | BL21(DE3) *△aspA△ldhA* | | This study |
| BT180 | BL21(DE3)_ale180[7 mM-acetic acid^T^] | | This study |
| BT240 | BL21(DE3)_ale60[4 mM-Acrylic acid/8 mM-acetic acidᵀ] | | This study |
| BT412 | BT240_ale172[18 mM-Acrylic acid/20 mM-acetic acidᵀ] | | This study |
| BT412A | BT412 derivative, BT412 *△panD△fumAfumBfumC* | | This study |
| BT412B | BT412 derivative, BT412 *△panD△fumA△fumB△fumC△acuI* | | This study |
| BT412C | BT412 derivative, BT412 *△ldhA* | | This study |
|  | Plamids | |  |
| pET-28a (+) | Km^R^, T7 *promoter, pBR322 origin* | | Tsingke |
| pET-*acs* | pET-28a (+) derivative; containing *acs* gene from *E. coli* | | This study |
| pET-*gltA* | pET-28a (+) derivative; containing *gltA* gene from *E. coli* | | This study |
| pET-*acnB* | pET-28a (+) derivative; containing *acnB* gene from *E. coli* | | This study |
| pET-*aceA* | pET-28a (+) derivative; containing *aceA* gene from *E. coli* | | This study |
| pET-*sdhb* | pET-28a (+) derivative; containing *sdhb* gene from *E. coli* | | This study |
| pET-*fumABC* | pET-28a (+) derivative; containing *fumABC* gene from *E. coli* | | This study |
| pET-*aspA* | pET-28a (+) derivative; containing *fumABC* gene from *E. coli* | | This study |
| pET-*panD* | pET-28a (+) derivative; containing *panD* gene from *E. coli* | | This study |
| pET-*act* | pET-28a (+) derivative; containing *act* gene from *A. propionicum* | | This study |
| pET-*acl2* | pET-28a (+) derivative; containing *acl2* gene from *A. propionicum* | | This study |
| pET-*yciA* | pET-28a (+) derivative; containing *yciA* gene from *E. coli* | | This study |
| pET-*dmlA* | pET-28a (+) derivative; containing *dmlA* gene from *E. coli* | | This study |
| pET-*ldh2* | pET-28a (+) derivative; containing *ldh2* gene from *E. coli* | | This study |
| pET-*ldhA* | pET-28a (+) derivative; containing *ldhA* gene from *E. coli* | | This study |
| pET-*panD^*^* | pET-28a (+) derivative; containing *panD^*^* (L20G/R99T) gene from *E. coli* | | This study |
| pET-*yciA^*^* | pET-28a (+) derivative; containing *yciA^*^* (D39T/S89D) gene from *E. coli* | | This study |
| pET-*dmlA^*^* | pET-28a (+) derivative; containing *dmlA^*^* (E99D/N293D) gene from *E. coli* | | This study |
| pET-*acs*-*panD* | pET-28a (+) derivative; containing *asc* gene from *E. coli, panD* gene from *E. coli* | | This study |
| pET-*acs*-*panD^*^* | pET-28a (+) derivative; containing *asc* gene from *E. coli, panD^*^* (L20G/R99T) gene from *E. coli* | | This study |
| pET-*acs*-*panD*-*act* | pET-28a (+) derivative; containing *acs* gene from *E. coli, panD* gene from *E. coli* and *act* gene from *A. propionicum* | | This study |
| pET-*acs*-*panD^*^*-*act* | pET-28a (+) derivative; containing *acs* gene from *E. coli, panD^*^* (L20G/R99T) gene from *E. coli* and *act* gene from *A. propionicum* | | This study |
| pET-*acs*-*panD*-*act*-*acl2* | pET-28a (+) derivative; containing *acs* gene from *E. coli*, *panD* gene from *E. coli*, *act* gene from *A. propionicum* and acl2 gene from *A. propionicum* | | This study |
| pET-*acs*-*panD^*^*-*act*-*acl2* | pET-28a (+) derivative; containing *acs* gene from *E. coli*, *panD^*^* (L20G/R99T) gene from *E. coli*, *act* gene from *A. propionicum* and acl2 gene from *A. propionicum* | | This study |
| pET-*acs*-*panD*-*act-acl2*-*yciA* | pET-28a (+) derivative; containing *acs* gene from *E. coli*, *panD* gene from *E. coli*, *act* gene from *A. propionicum*, acl2 gene from *A. propionicum* and *yciA* gene from *E. coli* | | This study |
| pET-*acs*-*panD^*^*-*act-acl2*-*yciA^*^* | pET-28a (+) derivative; containing *acs* gene from *E. coli*, *panD^*^* (L20G/R99T) gene from *E. coli*, *act* gene from *A. propionicum*, acl2 gene from *A. propionicum* and *yciA* (D39T/S89D) gene from *E. coli* | | This study |
| pET-*dmlA* | pET-28a (+) derivative; containing *dmlA* gene from *E. coli* | | This study |
| pET-*dmlA^*^* | pET-28a (+) derivative; containing *dmlA* (E99D/N293D) gene from *E. coli* | | This study |
| pET-*dmlA-ldh2* | pET-28a (+) derivative; containing *dmlA* gene from *E. coli* and *ldh2* gene from *E. coli* | | This study |
| pET-*dmlA^*^-ldh2* | pET-28a (+) derivative; containing *dmlA* (E99D/N293D) gene from *E. coli* and *ldh2* gene from *E. coli* | | This study |

**Supplementary Table 5. Synthetic pathways and required enzymes for C3 chemicals**

| **Name** | **Enzyme name and source** |
| --- | --- |
| Acrylic acid | Citrate synthase (*gltA*, *Escherichia coli*)  Aconitate hydratase（*acnB*，*Escherichia coli*）  Isocitrate lyase（*aceA*，*Escherichia coli* ）  Succinate dehydrogenase （*sdhb*， *Escherichia coli* ）  Aspartate ammonia-lyase（*aspA*，*Eschericia coli*）  Aspartate 1-decarboxylase（*panD*， *Eschericia coli*）  β-alanine CoA-transferase（*act*， *Anaerotignum propionicum*）  β-alanyl-CoA: ammonia lyase 2（*acl2*， *Anaerotignum propionicum*）  Acyl-CoA thioester hydrolase（*yciA*，*Escherichia coli*） |
| L-lactate acid | Citrate synthase (*gltA*, *Escherichia coli*)  Aconitate hydratase（*acnB*，*Escherichia coli*）  Isocitrate lyase（*aceA*，*Escherichia coli* ）  Succinate dehydrogenase （*sdhb*， *Escherichia coli* ）  Aspartate ammonia-lyase（*aspA*，*Eschericia coli*）  Aspartate 1-decarboxylase（*panD*， *Eschericia coli*）  Malate dehydrogenase (*dmlA*, *Eschericia coli)*  L-lactate dehydrogenase（*ldh2*，*Escherichia coli*） |
| β-alanine | Citrate synthase (*gltA*, *Escherichia coli*)  Aconitate hydratase（*acnB*，*Escherichia coli*）  Isocitrate lyase（*aceA*，*Escherichia coli* ）  Succinate dehydrogenase （*sdhb*， *Escherichia coli* ）  Aspartate ammonia-lyase（*aspA*，*Eschericia coli*）  Aspartate 1-decarboxylase（*panD*， *Eschericia coli*） |

**Supplementary Table 6. Composition analysis of fermentation broth**

| **Components** | **For β-alanine** | | **For Acrylic acid** | | **For L-lactic acid** | |
| --- | --- | --- | --- | --- | --- | --- |
|  | **Concentration (mg/L)** | | | | | |
| Acetyl-CoA | 0.78 | 0.0% | 0.56 | 0.1% | 0.72 | 0.1% |
| citrate | 4.95 | 0.2% | 2.45 | 0.3% | 1.64 | 0.2% |
| D-threo-isocitrate | 2.36 | 0.1% | 0.74 | 0.1% | 0.77 | 0.1% |
| Succinic | 7.78 | 0.3% | 1.75 | 0.2% | 2.31 | 0.3% |
| Glyoxylate | 6.94 | 0.3% | 0.78 | 0.1% | 2.34 | 0.3% |
| Fumarate | 122.21 | 5.0% | 0.04 | 0.0% | 22.22 | 2.7% |
| Aspartate | 180.84 | 7.1% | 3.27 | 0.4% | 9.85 | 1.2% |
| β-alanine | 2147.42 | 84.2% | 27.06 | 3.2% | 25.48 | 3.1% |
| β-alanyl-CoA | 0.00 | 0.0% | 1.26 | 0.2% | 0.00 | 0.0% |
| Acryly-CoA | 0.00 | 0.0% | 1.32 | 0.2% | 0.00 | 0.0% |
| Acrylic acid | 0.00 | 0.0% | 752.43 | 92.4% | 0.00 | 0.0% |
| Malate | 0.12 | 0.0% | 0.06 | 0.0% | 17.03 | 2.1% |
| Pyruvate | 5.46 | 0.2% | 0.42 | 0.1% | 34.57 | 4.2% |
| L-lactic acid | 3.94 | 0.2% | 0.05 | 0.0% | 672.88 | 81.8% |
| D-lactic acid | 0.01 | 0.0% | 0.01 | 0.0% | 0.0 | 0.0% |
| Inorganic salt | 67.57 | 1.4% | 22.09 | 2.7% | 32.78 | 2.8% |

Component analysis was performed by the Analytical and Testing Center of Chongqing University.

**Supplementary Table 7. Comparison of product yields with other related literature**

| **Substrates** | **Product** | **g/g** | **Sources** |
| --- | --- | --- | --- |
| Acetic acid | n-Butanol | 0.26 | ^1^ |
| Acetic acid | Amorphadiene | 0.25 | ^1^ |
| Acetic acid | Epi-Aristolochene | 0.11 | ^1^ |
| Acetic acid | Casinene | 0.05 | ^1^ |
| Acetic acid | PHB | 0.52 | ^1^ |
| Acetic acid | PHB | 0.75 | ^2^ |
| Acetic acid | Glucose | - | ^3^ |
| CO_2_ | Butanol and hexanol | 0.40 | ^4^ |
| Methanol | Ethylene glycol | 0.56 | ^5^ |
| Methanol | Glycolic acid | 0.21 | ^5^ |
| Methanol | D-erythrose | 0.034 | ^5^ |
| Acetic acid | β-alanine | 0.86 | This study |
| Acetic acid | Acrylic acid | 0.84 | This study |
| Acetic acid | L-lactic acid | 0.47 | This study |

**Supplementary Table 8. Key genes and its sequences**

| **Name** | **Sequences** |
| --- | --- |
| *panD* | 5`- ATGATTCGCACGATGCTGCAGGGCAAACTCCACCGCGTGAAAGTGACTCATGCGGACCTGCACTATGAAGGTTCTTGCGCCATTGACCAGGATTTTCTTGACGCAGCCGGTATTCTCGAAAACGAAGCCATTGATATCTGGAATGTCACCAACGGCAAGCGTTTCTCCACTTATGCCATCGCGGCAGAACGCGGTTCGAGAATTATTTCTGTTAACGGTGCGGCGGCCCACTGCGCCAGTGTCGGCGATATTGTCATCATCGCCAGCTTCGTTACCATGCCAGATGAAGAAGCTCGCACCTGGCGACCCAACGTCGCCTATTTTGAAGCGACAATGAAATGAAACGTACCGCGAAAGCGATTCCGGTACAGGTTGCTTGA -3` |
| *yciA* | 5`- ATGTCTACAACACATAACGTCCCTCAGGGCGATCTTGTTTTACGTACTTTAGCCATGCCCGCCGATACCAATGCCAATGGTGACATCTTTGGTGGTTGGTTAATGTCACAAATGGATATTGGCGGCGCTATTCTGGCAAAAGAAATTGCCCACGGTCGCGTAGTGACTGTGCGGGTTGAAGGAATGACTTTCTTACGGCCGGTTGCGGTCGGCGATGTGGTGTGCTGCTATGCACGCTGTGTCCAGAAAGGGACGACATCGGTCAGCATTAATATTGAAGTGTGGGTGAAAAAAGTAGCGTCTGAACCAATTGGGCAACGCTATAAAGCGACAGAAGCATTATTTAAGTATGTCGCGGTTGATCCTGAAGGAAAACCTCGCGCCTTACCTGTTGAGTAA -3` |
| *dmlA* | 5`- ATGATGAAAACGATGCGTATTGCTGCGATCCCGGGAGACGGGATTGGCAAAGAAGTCCTTCCTGAAGGGATTCGCGTGTTACAGGCTGCCGCTGAGCGCTGGGGCTTCGCCTTGAGTTTTGAGCAAATGGAGTGGGCGAGCTGCGAGTATTACAGCCATCACGGTAAAATGATGCCGGACGACTGGCATGAGCAACTTAGCCGTTTCGACGCCATCTATTTTGGTGCCGTCGGCTGGCCGGATACCGTTCCGGACCATATTTCGTTGTGGGGTTCGCTGCTGAAATTTCGTCGTGAATTCGACCAGTACGTCAACCTGCGCCCGGTTCGTCTCTTTCCTGGCGTTCCCTGCCCGCTGGCGGGAAAACAGCCTGGCGACATCGATTTTTACGTGGTCAGGGAAAACACCGAAGGCGAATATTCCTCGCTCGGCGGTAGAGTGAATGAAGGTACAGAGCATGAAGTCGTCATTCAGGAATCGGTATTTACCCGCCGTGGTGTCGATCGCATTTTGCGTTATGCCTTCGAACTTGCGCAAAGCCGTCCACGTAAGACACTAACTTCTGCCACTAAATCGAACGGTTTAGCCATCAGCATGCCGTACTGGGATGAGCGAGTGGAAGCAATGGCCGAGAATTACCCGGAGATCCGCTGGGACAAGCAGCATATTGATATTCTCTGCGCGCGTTTTGTGATGCAGCCGGAACGATTCGATGTGGTGGTGGCGTCCAATTTGTTTGGCGATATCCTTTCCGATCTTGGCCCGGCCTGCACCGGCACCATTGGCATTGCCCCATCCGCCAACCTGAATCCGGAACGCACTTTCCCGTCGCTCTTCGAGCCTGTCCACGGTTCCGCGCCGGATATCTACGGGAAAAATATTGCTAACCCTATCGCCACGATTTGGGCCGGGGCAATGATGCTCGATTTTCTCGGCAATGGCGATGAGCGTTTCCAGCAAGCGCATAACGGTATTCTGGCAGCAATTGAAGAAGTGATTGCTCACGGGCCGAAAACACCTGATATGAAAGGCAATGCCACCACGCCACAGGTTGCCGACGCGATTTGCAAAATTATTTTGCGTTAA -3` |

**Supplementary Table 9. Kinetic parameters of Aspartate 1-decarboxylase (ADC), Acyl-CoA thioester hydrolase (ACH) and Malate dehydrogenase (MDH) enzymes**

| **Enzymes** | **K_m_(mM)** | **K_cat_** | **Specificity^a^ (U/mg)** |
| --- | --- | --- | --- |
| ADC^#^ (L20E/R99T) | 2.34 ±0.07 | 0.12 ± 0.01 s^-1^ | 15.58 ± 1.14 |
| ADC^#^ (L20G/R99T) | 2.45 ± 0.08 | 0.68 ± 0.03 s^-1^ | 132.24 ± 6.17 |
| ADC^#^ (N112E/V123D) | 2.18 ± 0.06 | 0.16 ± 0.01 s^-1^ | 24.66 ± 1.87 |
| ADC^#^ (N112A/V123D) | 2.09 ± 0.06 | 0.37 ± 0.02 s^-1^ | 75.46 ± 6.24 |
| ACH^#^ (D39E/S89D) | 3.72 ± 0.11 | 0.16 ± 0.01 s^-1^ | 15.93 ± 0.92 |
| ACH^#^ (D39A/S89D) | 3.99 ± 0.11 | 0.88 ± 0.03 s^-1^ | 46.21 ± 3.57 |
| ACH^#^ (D39T/S89D) | 4.28 ± 0.11 | 1.47 ± 0.06 s^-1^ | 99.75 ± 4.31 |
| ACH^#^ (D39L/S89D) | 4.05 ± 0.12 | 0.84 ± 0.03 s^-1^ | 44.21 ± 3.28 |
| MDH^#^ (N135G/R183D) | 6.87 ± 0.25 | 1.82 ± 0.05 s^-1^ | 15.67 ± 0.84 |
| MDH^#^ (N135K/R183D) | 6.47 ± 0.18 | 3.04 ± 0.11 s^-1^ | 25.25 ± 1.16 |
| MDH^#^ (E99K/N293D) | 6.92 ± 0.27 | 2.71 ± 0.09 s^-1^ | 28.47 ± 1.27 |
| MDH^#^ (E99D/N293D) | 7.2 ± 0.21 | 6.30 ± 0.21 s^-1^ | 42.64 ± 2.84 |

Data are presented as means ± STDV calculated from at least three replicates.

^a^Specificity of Aspartate 1-decarboxylase (ADC^#^) refers to the specificity of aspartate to β-alanine.

^a^Specificity of Acyl-CoA thioester hydrolase (ACH^#^) refers to the specificity of acrylyl-CoA to acrylic acid.

^a^Specificity of malate dehydrogenase (MDH) refers to the specificity of malate to pyruvate.

**Supplementary Table 10. Comparison of this work with representative electrocatalytic CO_2_ to acetate systems.**

| **Catalyst** | **Biocompatibility (electrolyte and reaction conditions)** | **Faradaic efficiency** | **Byproducts** | **Duration (h)** | **Acetate production rate** | **Reference** |
| --- | --- | --- | --- | --- | --- | --- |
| Cu-Ag tandem electrocatalyst | 0.1 M CsH_2_PO_4_ or KH_2_PO_4_ | 21% | CO, ethylene, formate， ethanol, n-propanol, and H_2_ | 129 | ~1.55 mM h^-1^ | ^6^ |
| *sp*^2^ /*sp*^3^ hybridization carbon catalyst | 0.1 M KOH | 62.7% | Formate, CO, and H_2_ | 100 | N. A. | ^7^ |
| Ni-N-C single atom catalyst for CO_2_ to CO，and GB-Cu for CO to acetate | 1 M KOH (CO_2_ to CO) and solid-electrolyte (CO_2_ to acetate) | 46% | Ethanol,  n-propanol,  ethylene, and  H_2_ | 140 | 3.69 mM h^-1^ | ^8^ |
| PcNi-DMTP (COF) for CO_2_ to CO，and MAF-2 (MOF) for CO to acetate | 1 M KOH | 51.2% | Methanol, formate, methane, ethylene, ethanol, propanol, CO, and H_2_ | 200 | 0.1 mM h^-1^ | ^9^ |
| Cu/CuO*_x_* | 6 M KOH and 0.8 M H_3_BO_3_; 58 atm CO_2_ (g) | 87% | Formate, ethanol, ethylene, methane, CO, and H_2_ | 20 | 30 mg h^-1^ cm^-2^ | ^10^ |
| Stainless steel mesh/Co-P | DSMZ 311 medium | 71.46% ± 2.75 | H_2_ | 120 | 0.24 mM h^-1^ | This study |

**Table S11.** **Comparison of production cost with traditional method.**

| **Module** | **Items** | **β-alanine ($/ton)** | | **L-lactic acid ($/ton)** | | **Acrylic acid ($/ton)** | |
| --- | --- | --- | --- | --- | --- | --- | --- |
|  |  | **Traditional method^11^** | **This study** | **Traditional method^12^** | **This study** | **Traditional method^13^** | **This study** |
| Microbial electroreduction | CO_2_ | / | 16.32 | / | 21.76 | / | 17.28 |
|  | Nitrogen source | / | 0.62 | / | 0.82 | / | 0.65 |
|  | Energy | / | 1108 | / | 1477 | / | 1173 |
|  | Separation and purification | / | 60 | / | 80 | / | 63.5 |
| Biosynthesis | Carbon source | 143 | 0 | 241 | 0 | 210 | 0 |
|  | Nitrogen source | 255 | 278 | 156 | 372 | 265 | 296 |
|  | Energy | 166 | 177 | 184 | 407 | 250 | 577 |
|  | Separation and purification | 199 | 106 | 148 | 122 | 100 | 116 |
| **Total** | | **763** | **1746** | **729** | **2481** | **^#^** | **2243** |

/ Indicates that the item is not provided in the literature or not required.

The calculations in the table are based on the cost required to produce one ton of product, with the price of electricity set at $0.05 per kWh.


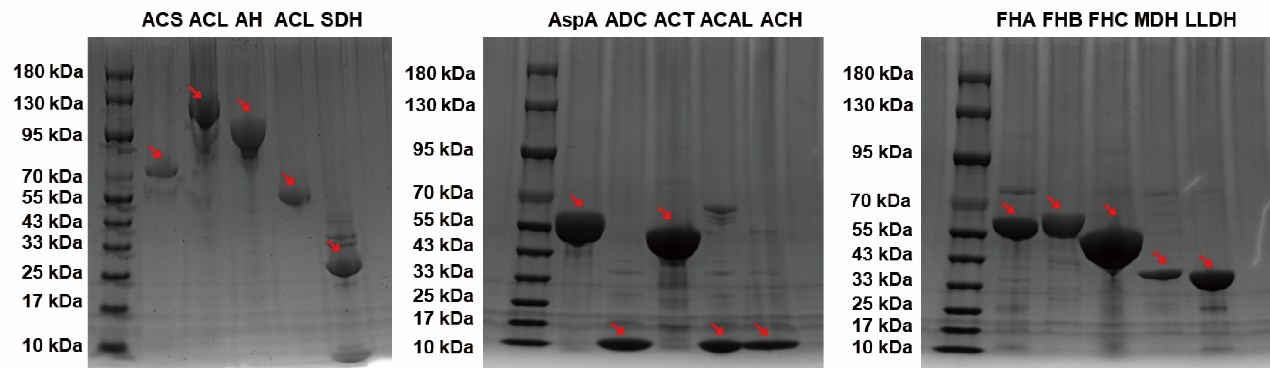


**Fig. S1. Expression and purification of enzymes involved in the synthesis part.** ACS, Acetyl-coenzyme A synthetase; CS, citrate synthase; SDH, Succinate dehydrogenase; FH, Fumarate hydratase; AspA, Aspartate ammonia-lyase; ADC, Aspartate 1-decarboxylase; ACT, β-alanine CoA-transferase; ACAL, β-alanyl-CoA: ammonia lyase; ACH, CoA thioesterase; MDH, malate dehydrogenase; DLDH, D-lactate dehydrogenase; LLDH, L-lactate dehydrogenase.


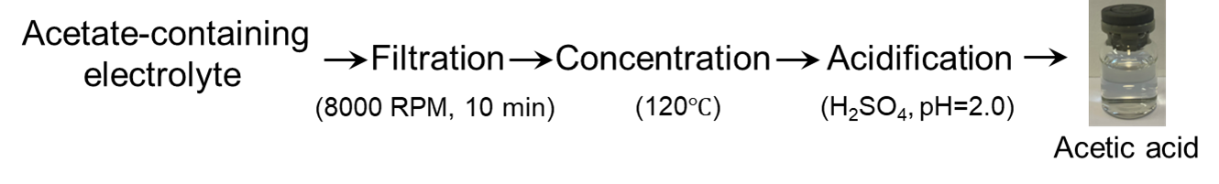


**Fig. S2. Recovery and Purification of Acetic acid.** Acetic acid was recovered from module I. First, the acetate-containing electrolyte was centrifuged (8000 RPM, 10 minutes) and filtered to remove biological cells and insoluble substances. Subsequently, the electrolyte was concentrated to 20% of its initial volume by solvent evaporation at 120°C. The pH was then acidified to 2.0 using concentrated sulfuric acid to convert acetate to acetic acid.


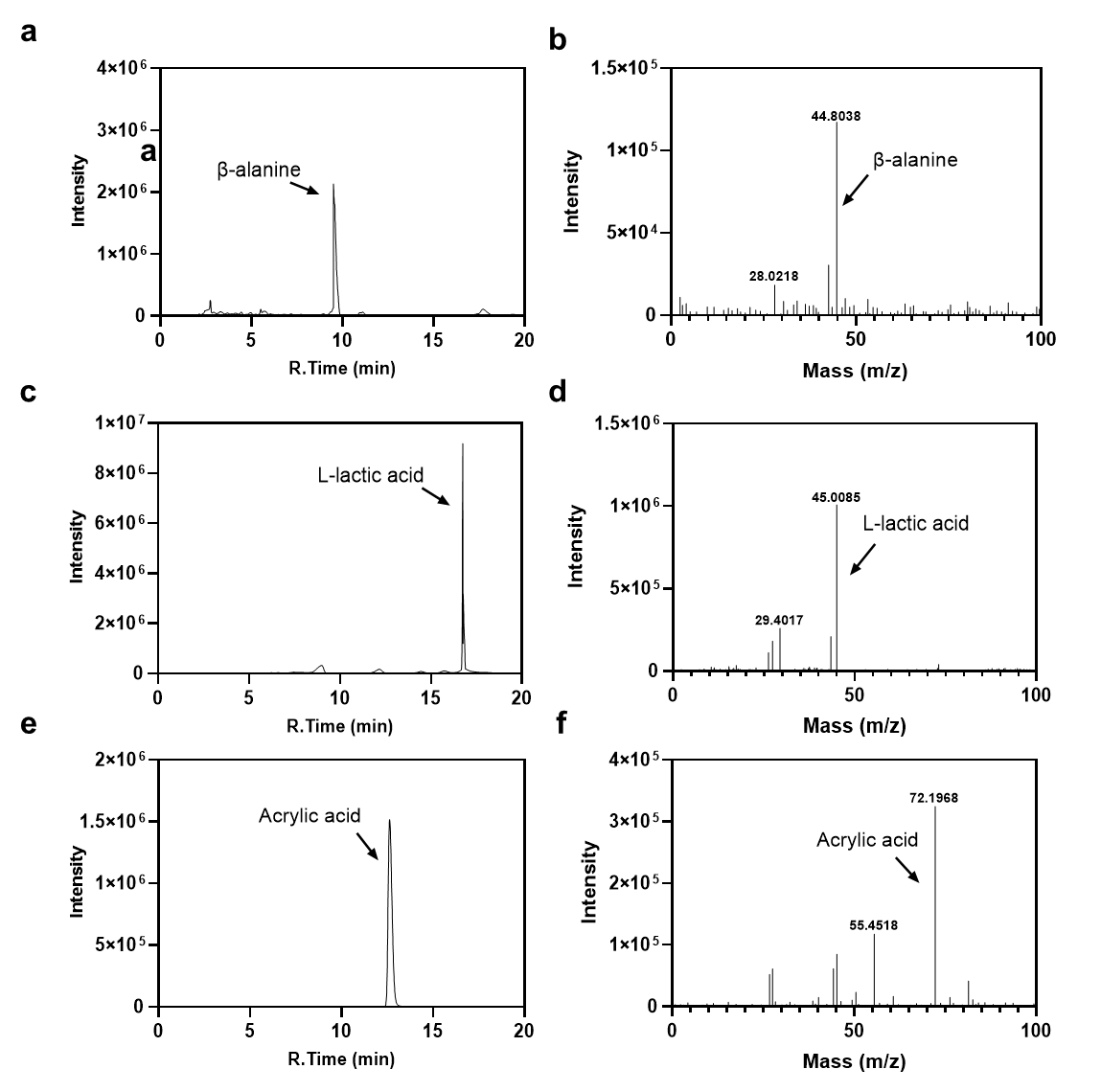


**Fig. S3. LC-MS confirmation of β-alanine, L-lactic acid and acrylic acid.** a. HPLC results of β-alanine from fermentation broth. b. Mass spectrum of β-alanine from fermentation broth. c. HPLC results of L-lactic acid from fermentation broth. d. Mass spectrum of L-lactic acid from fermentation broth. e. HPLC results of acrylic acid from fermentation broth. e. Mass spectrum of acrylic acid from fermentation broth.


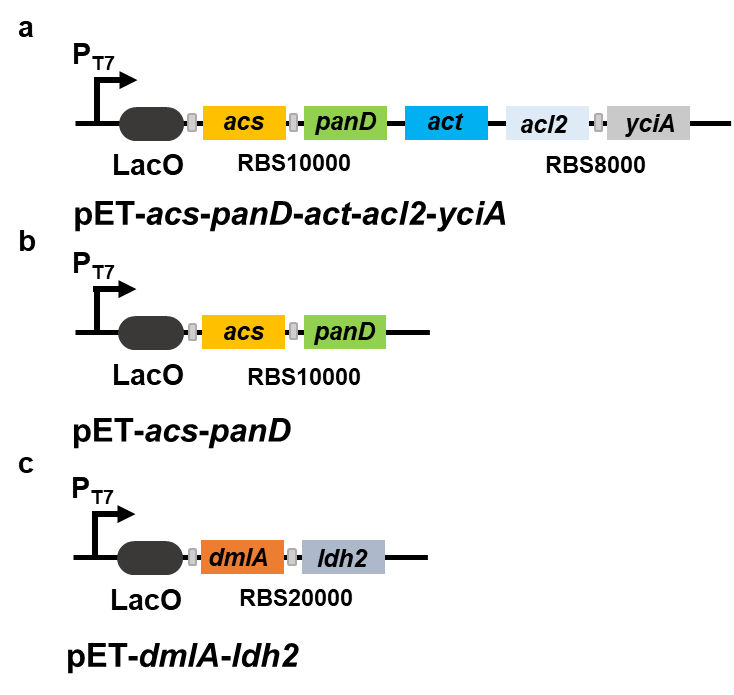


**Fig. S4. Expression of genes in the plasmid of the fermentation strains.**

a. Addition of RBS binding sites with varying strengths upstream of the panD and yciA genes, respectively. b. Addition of an RBS binding site upstream of the panD gene. c. Addition of an RBS binding site upstream of the Ldh2 gene. The entire plasmid expression cassette utilizes the native RBS from the pET28a vector.


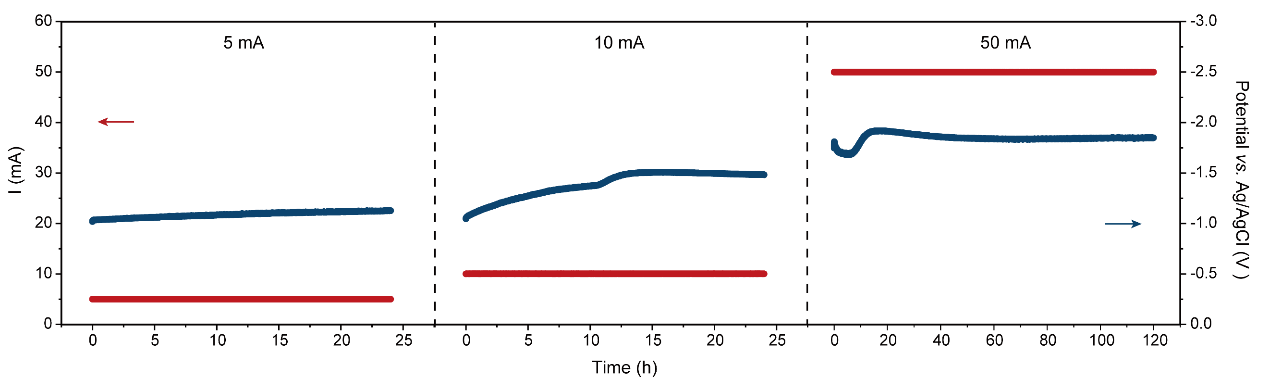


**Fig. S5**. Time-dependent current and electrode potential at different galvanostatic process.


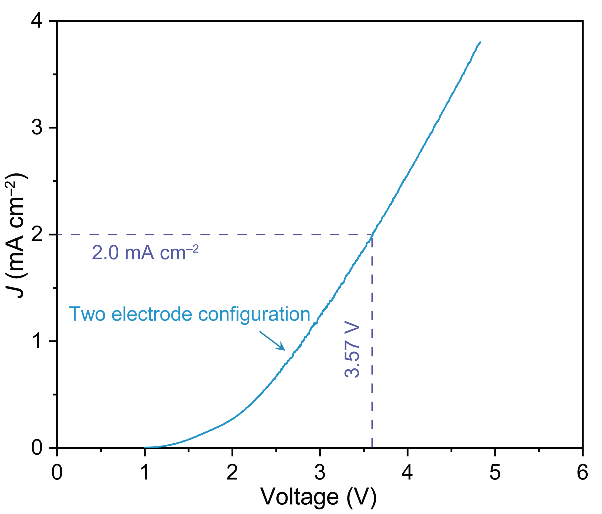


**Fig. S6**. J-V curve of the MES in a two-electrode configuration.

**Supplementary Method 1: Energy consumption calculation method**

An electricity price of 0.05 USD/kWh was used for all operational cost calculations.

The total current required for the device is calculated as follows:

2 mA/cm^2^ × 25 cm^2^ × 5 days × 24 h/day ÷ 1000 = 6 A·h

With a battery voltage of 3.2 V, the energy consumption is:

6 A·h × 3.2 V ÷ 1000 = 0.019 kW·h = 6.9 × 10^4^ J

Based on atomic conservation, the conversion of one ton of CO_2_ yields 0.68 tons of acetic acid. The previous calculation indicated an energy consumption of 6.9×10^4^ J for producing 1.2 g of acetic acid. Therefore, the energy required to produce 0.68 tons of acetic acid would be 39.1 GJ.

Using a 100 L-bioreactor for subsequent biosynthesis calculation then. For 100 L bioreactor be operated for once, the energy consumption is estimated as 1 kWh. For alanine production, 100 L-bioreactor produced 138 g product. So production of 1 ton of alanine cost 26.1 GJ. For acrylic acid production, 100 L-bioreactor produced 45 g product. So production of 1 ton of acrylic acid cost 80.0 GJ. For L-lactic acid production, 100 L-bioreactor produced 40 g product. So production of 1 ton of L-lactic acid cost 90.0 GJ.

The energy consumption for converting 0.68 tons of acetic acid into various C3 compounds is calculated as follows:

For alanine (conversion ratio 1:0.72):

26.1 × 0.68 ×0.72 = 12.78 GJ

For acrylic acid (conversion ratio 1:0.54):

80.0 × 0.68 ×0.54 = 29.3 GJ

For L-lactic acid (conversion ratio 1:0.68):

90.0 × 0.68 ×0.68 = 41.6 GJ

**Supplementary References**

1. Liu, C., Gallagher, J.J., Sakimoto, K.K. et al., Nanowire–Bacteria Hybrids for Unassisted Solar Carbon Dioxide Fixation to Value-Added Chemicals, *Nano Letters* (2015): 3634-3639. https://doi.org/10.1021/acs.nanolett.5b01254.

2. Lee, G., Jo, H.-J., Choi, J. et al., CO_2_ upgrading into bioproducts using a two-step abiotic–biotic system, *Proceedings of the National Academy of Sciences* (2025): e2512565122. https://doi.org/10.1073/pnas.2512565122.

3. Zheng, T., Zhang, M., Wu, L. et al., Upcycling CO_2_ into energy-rich long-chain compounds via electrochemical and metabolic engineering, *Nature Catalysis* (2022): 388-396. https://doi.org/10.1038/s41929-022-00775-6.

4. Haas, T., Krause, R., Weber, R., Demler, M. & Schmid, G., Technical photosynthesis involving CO_2_ electrolysis and fermentation, *Nature Catalysis* (2018): 32-39. https://doi.org/10.1038/s41929-017-0005-1.

5. Zhou, J., Tian, X., Yang, Q. et al., Three multi-enzyme cascade pathways for conversion of C1 to C2/C4 compounds, *Chem Catalysis* (2022): 2675-2690. https://doi.org/10.1016/j.checat.2022.07.011.

6. Lee, G., Jo, H.-J., Choi, J. et al., CO_2_ upgrading into bioproducts using a two-step abiotic–biotic system, *Proceedings of the National Academy of Sciences* (2025): e2512565122. https://doi.org/10.1073/pnas.2512565122.

7. Wang, C., Zhang, G., Luo, R. et al., Selective CO_2_ reduction to acetate via controlled sp 2/sp 3 carbon hybridization, *Nature Communications* (2025): 10506. http://doi.org/10.1038/s41467-025-65504-6.

8. Zheng, T., Zhang, M., Wu, L. et al., Upcycling CO_2_ into energy-rich long-chain compounds via electrochemical and metabolic engineering, *Nature Catalysis* (2022): 388-396. https://doi.org/10.1038/s41929-022-00775-6.

9. Zhu, H.-L., Huang, J.-R., Zhang, M.-D. et al., Continuously producing highly concentrated and pure acetic acid aqueous solution via direct electroreduction of CO_2_, *Journal of the American Chemical Society* (2024): 1144-1152. http://doi.org/10.1021/jacs.3c12423.

10. Li, J., Kuang, Y., Zhang, X. et al., Electrochemical acetate production from high-pressure gaseous and liquid CO_2_, *Nature Catalysis* (2023): 1151-1163. http://doi.org/:10.1038/s41929-023-01046-8.

11. Ma, Y., Guo, X., Han, M. et al., Direct Synthesis of Amino Acids from Plastic, Air, and Water, *Angewandte Chemie* (2025): e202511466. http://doi.org/10.1002/anie.202511466.

12. Yu, W., Zhang, C., Li, Y. et al., Methanol biotransformation for the production of biodegradable plastic monomer L-lactate in yeast, *Nature Communications* (2025): 10756. http://doi.org/10.1038/s41467-025-65793-x.

13. Ko, Y.-S., Kim, J.W., Chae, T.U., Song, C.W. & Lee, S.Y., A Novel Biosynthetic Pathway for the Production of Acrylic Acid through β-Alanine Route in Escherichia coli, *ACS Synthetic Biology* (2020): 1150-1159. http://doi.org/10.1021/acssynbio.0c00019.
